# Integrating smoking cessation into HIV care settings: A systematic review and meta-analysis of effectiveness and the evidence gap in cost-effectiveness

**DOI:** 10.1371/journal.pone.0350040

**Published:** 2026-07-30

**Authors:** Van Minh Nguyen, Thanh Hoang, Yesim Tozan, Mikael Svensson, Minh Van Hoang, Nawi Ng

**Affiliations:** 1 School of Public Health and Community Medicine, Institute of Medicine, Sahlgrenska Academy, University of Gothenburg, Gothenburg, Sweden; 2 Hanoi University of Public Health, Hanoi, Vietnam; 3 New York University School of Global Public Health, Department of Global and Environmental Health, New York University, New York, United States of America; University of Perugia Department of Medicine: Universita degli Studi di Perugia Dipartimento di Medicina, ITALY

## Abstract

The prevalence of smoking among people living with HIV (PLWH) is higher than in the general population, and PLWH who smoke are at increased risk of both smoking- and HIV-related comorbidities. As most PLWH reside in low- and middle-income countries (LMICs), there is a need for effective and cost-effective smoking cessation interventions in resource-constrained settings. We systematically reviewed and meta-analyzed the effectiveness of smoking cessation interventions for PLWH and looked for economic evaluations. Four databases (PubMed, Cochrane, Scopus, Web of Science) were searched up to March 23^rd^, 2026. Interventional and quasi-experimental studies evaluating smoking cessation interventions for PLWH were included. Risk of bias was assessed using Cochrane’s risk-of-bias tool for randomized studies and the Effective Public Healthcare Panacea Project tool for non-randomized studies. Thirty-two articles met the inclusion criteria. Most evidence originated from high-income countries, with three randomized controlled trials conducted in LMICs (Kenya, South Africa and Vietnam). No economic evaluations were identified. Smoking cessation interventions varied in type, duration, intensity, and mode of delivery. Overall, studies had low to moderate risk of bias. GRADE assessments indicated moderate-certainty evidence that pharmacological interventions (RR 1.86, 95% CI 1.42–2.45) and tailored, intensive behavioral support (RR 1.34, 95% CI 1.05–1.71) increase smoking abstinence compared with standard care. This review indicates that pharmacological and tailored, intensive behavioral support interventions can support smoking cessation among PLWH, including emerging evidence from LMICs, but the absence of economic evaluations limits guidance for resource-constrained settings. Future research should prioritise implementation strategies and economic evaluations to support scalable integration into routine HIV care. (PROSPERO Registration no: CRD42022313630).

## Introduction

Tobacco use is a major public health challenge, causing more than 7 million deaths annually [1]. Smoking tobacco is substantially more harmful to people living with human immunodeficiency virus (HIV), posing a greater threat to long-term survival than the HIV infection itself [2, 3]. A study of people living with HIV (PLWH) in Europe and North America showed that participants who smoked had a 1.94-fold higher mortality rate compared to those who never smoked. This risk was particularly elevated for cardiovascular diseases and non-AIDS cancers, with mortality rate ratios of 6.28 and 2.67, respectively [2]. Despite being more vulnerable to the adverse health effects of smoking, a 2021 systematic review pointed out that PLWH were 64% more likely to smoke than the general population [4].

The burden of smoking among PLWH is more significant in low- and middle-income countries (LMICs), where populations face a double burden of high smoking and HIV prevalences. On one hand, 79% of global smokers reside in LMICs, with the average adult smoking prevalence estimated at 15.6% in middle-income countries and 10.3% in low-income countries [5]. However, the smoking prevalence among men living with HIV in LMICs was estimated at 24.4%, a rate significantly higher than among HIV-negative men (risk ratio: 1.46, 95% confidence interval: 1.30 to 1.65) [6]. This pronounced gender disparity is largely attributed to societal norms and gender inequalities that stigmatize tobacco use among women while socially accepting it among men [7]. Furthermore, the elevated smoking prevalence across the broader PLWH population is driven by high rates of co-occurring substance use and the reliance on tobacco as a coping mechanism for HIV-related psychological distress, including depression, stigma, and loneliness [7]. On the other hand, the global HIV burden is predominantly borne by LMICs, accounting for 36.94 million PLWH and 705,800 HIV-related deaths, compared to just 3.13 million PLWH and 12,200 deaths in high-income countries (HICs) [8]. The disproportionate HIV burden in LMICs is driven by factors such as low socioeconomic status and limited healthcare access, which have facilitated the spread of the virus in these settings [9].

The combination of high smoking prevalence and HIV burden in LMICs, coupled with the adverse health effects of smoking on PLWH, underscore the urgent need for effective and cost-effective smoking cessation interventions tailored to PLWH in these resource-constrained settings. However, the evidence base evaluating these strategies remains heavily dominated by research conducted in HICs [10–12]. A 2024 Cochrane systematic review synthesizing the global evidence base highlighted this geographical disparity, noting that the literature is predominantly from the United States, which limits global generalizability [12]. Out of the 17 studies included in the review, only one was conducted in LMIC (South Africa), for which the reviewers had to rely on unpublished data provided directly by the trial investigators. This severe geographic disparity led the authors to emphasize a critical need for further research in resource-constrained contexts facing a high dual burden of HIV and tobacco consumption [12].

Since the 2024 Cochrane review, several additional studies have been published, including trials from LMICs and the first trial to evaluate bupropion in this population. An updated synthesis is therefore needed to assess whether the evidence base has changed and to clarify how emerging LMIC evidence compares with the HIC-dominated literature. This systematic review and meta-analysis aimed to synthesize the effectiveness of smoking cessation interventions for PLWH and to assess whether cost or cost-effectiveness evidence is available to guide implementation, particularly in resource-constrained settings. To address the LMIC evidence gap directly, we report LMIC studies distinctly and interpret them alongside the global evidence.

## Methods

This systematic review and meta-analysis was conducted and reported in accordance with the Preferred Reporting Items for Systematic Reviews and Meta-Analyses (PRISMA) 2020 statement [13] (See S1 Checklist). The study protocol was registered with PROSPERO (Registration no: CRD42022313630). Because the specific interventions could not be anticipated when the protocol was registered, the comparison groupings used for synthesis were defined post hoc from the clinical and methodological characteristics of the included studies. Further methods are also detailed in S1 Text.

### Inclusion criteria

We included peer-reviewed original research articles published in English and available in full text, with no restrictions on publication date. Eligible studies had to be randomized or non-randomized intervention studies providing primary data on the effectiveness and/or cost-effectiveness of smoking cessation interventions targeted at adult PLWH who smoke. In this review, non-randomized studies included quasi-experimental, cohort, observational, and single-arm intervention studies without individual random allocation. Studies were required to have a minimum 6-month follow-up, consistent with the Russell Standard for smoking cessation trials and with the 2024 Cochrane review, to capture clinically meaningful long-term cessation [12, 14]. Review articles were excluded.

### Search strategy

The search strategy was developed using the PICO framework [15] and incorporated both MeSH terms (medical subject headings) and non-MeSH search terms, as shown in Box 1. The initial search terms were developed for PubMed and subsequently adapted to other databases using Polyglot Search Translator [16]. Four electronic databases (PubMed, Cochrane, Scopus, and Web of Science) were searched on March 23^rd^, 2026. Manual search was also conducted on the reference lists of included articles. The full search strategy is provided in S1 Table.

**Box 1. PICO framework.**

**Table:** 

Population	“People living with HIV”
Intervention	“Smoking cessation”
Comparator	Not restricted
Outcomes	“Smoking abstinence”, “cost”, “cost-effectiveness”

### Screening process

After removing duplicates using the de-duplication steps outlined by Bramer et al, two reviewers (VMN, TH) independently screened articles by title and abstract in Rayyan QCRI platform based on the inclusion criteria [17, 18]. They reached consensus on the articles for full-text review, with any disagreements resolved through discussion.

### Outcome measures

The primary outcome was smoking abstinence at six months or longer, preferably biochemically confirmed continuous abstinence or 7-day point-prevalence abstinence (PPA). We also sought economic outcomes for smoking cessation interventions for PLWH, expressed as the incremental cost-effectiveness ratio (ICER), with quality-adjusted life years (QALYs) or disability-adjusted life years (DALYs).

The secondary outcome measures of interest were changes in participants’ smoking behaviors (e.g., number of cigarettes smoked daily, number of quit smoking attempts, level of nicotine dependence), pre- and post-intervention, as well as improvements in quality of life, and changes in the risk of developing smoking-related illnesses (e.g., cardiovascular diseases, stroke and lung cancer).

### Risk of bias assessment

The risk of bias for all included articles was assessed independently by two authors (VMN and TH). For randomized studies, we used the *Cochrane risk-of-bias tool for randomized trials (RoB 2)* [19]. The tool provides a framework for evaluating the risk of bias in the study findings across five key domains: The randomization process, Deviations from the intended interventions, Missing outcome data, Outcome measurement, and Selection of the reported results. Each domain was graded using a three-level scale: Low risk of bias, Some concerns, High risk of bias [19]. For non-randomized studies, we used the *Effective Public Health Practice Project (EPHPP)’s Quality assessment tool for quantitative studies* [20]. The EPHPP tool assessed risk of bias using three levels: Strong, Moderate or Weak, where “Strong” indicates a low risk of bias and “Weak” indicates a high risk of bias. The tool consists of six domains (or “components”): Selection bias, Study design, Confounders, Blinding, Data collection method, and Withdrawals and dropouts [20].

### Data analysis and synthesis

Two reviewers (VMN and TH) independently extracted data from the included articles using a standardized Microsoft Excel form. Extracted information included study country, study design, intervention and comparator characteristics, participant characteristics, outcome definitions, follow-up duration, and abstinence outcomes. For the primary outcome we used intention-to-treat data where reported, otherwise, we calculated the risk ratios (RRs) from the original sample size and the number of abstinent participants at the relevant follow-up. Randomized trials formed the basis of the meta-analyses, whereas non-randomized studies were synthesized narratively and interpreted as lower-certainty evidence because they primarily informed real-world implementation, longer-term outcomes, and feasibility rather than causal efficacy. For studies with more than two study arms, eligible interventions were combined or compared using prespecified rules to avoid double-counting participants: multi-arm trials had their active arms pooled against the shared control; factorial trials had the main effect isolated by pooling across the other factor; and head-to-head and SMART trials informed direct comparative analyses rather than intervention-versus-control pooling. Full details of these rules are provided in S1 Text. Where quantitative synthesis was appropriate, effect sizes were pooled as RRs using a random-effects model with the generic inverse-variance method [15]. Between-study variance was estimated using the iterative Paule–Mandel estimator [21]. Sensitivity analyses compared the primary Paule–Mandel random-effects model with a fixed-effect model and the Hartung–Knapp–Sidik–Jonkman adjustment [22]. All analyses were performed in Python (version 3.11.14) using the statsmodels and PythonMeta packages; forest and funnel plots were generated with matplotlib.

### Assessment of the certainty of evidence (GRADE)

The certainty of evidence for each outcome was assessed using the GRADE approach [15, 23]. Randomized studies started as high-certainty evidence and non-randomized studies started as low-certainty evidence. Certainty was downgraded for risk of bias, inconsistency, indirectness, imprecision, and publication bias, and upgraded when a large effect was observed in the absence of serious limitations or plausible residual confounding. Publication bias was formally assessed only for comparisons including at least 10 studies. Final certainty was categorized as high, moderate, low, or very low. Full GRADE domain definitions and decision rules are provided in S1 Text.

## Results

**Fig 1:**
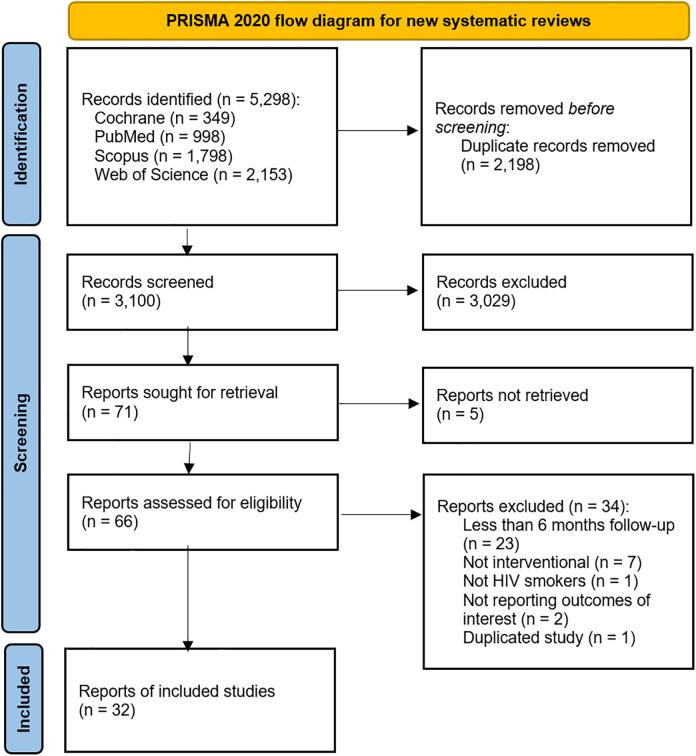
PRISMA 2020 flow diagram of study selection.

Our search yielded a total of 5,298 articles from four databases. After removing 2,198 duplicates, the titles and abstracts of 3,100 articles were screened. Fig 1 presents the PRISMA 2020 flow diagram of the different stages of study selection, including the reasons for study exclusion. A total of 71 articles were included for full-text review, and 66 were reviewed as the full-texts for 5 articles were unavailable. Thirty-four full-text articles were excluded due to the following reasons: short follow-up duration (i.e., less than 6 months, n = 23), no specific smoking cessation intervention provided (n = 7), outcomes not reported for people with HIV (n = 1), no relevant outcomes reported (n = 2), results from the same study were reported (n = 1). Ultimately, 32 articles were included in the systematic review. Detailed characteristics of the included studies are provided in S2 Table.

Among the excluded articles, four focused on smoking cessation interventions for PLWH in LMICs. These were excluded due to their short follow-up durations of 1 month [24], 2 months [25], 3 months [26] and 12 weeks [27]. Details of these studies are provided in S3 Table.

### Characteristics of included studies

Thirty-two articles reported smoking abstinence and related secondary outcomes; none evaluated the cost or cost-effectiveness of smoking cessation interventions for PLWH. Twenty-eight studies were conducted in HICs, including the USA (n = 17) [28–44], Canada (n = 2) [45, 46], New Zealand (n = 1) [47], and eight studies across seven European countries [48–55]. Sixteen studies were randomized controlled trials (15 parallel-group [28, 31–39, 42–44, 48, 55] and one factorial [41]) and 12 were non-randomized studies [29, 30, 40, 45–47, 49–54].

Four articles reported three randomized controlled trials conducted in LMICs, comprising 1,533 participants from Kenya, South Africa, and Vietnam. One trial was conducted in Nairobi, Kenya [56] (n = 300), one in Matlosana, South Africa [57, 58] (n = 561) and one in Hanoi, Vietnam [59] (n = 672). The South African evidence was reported in two articles: the original trial and a subsequent re-treatment extension [57, 58]. Set in urban and peri-urban HIV care settings, these trials enrolled predominantly male populations (range: 71% to 96%), with high engagement in HIV care (67% to 100% on antiretroviral therapy) [56–59]. Collectively they evaluated pharmacological and behavioral strategies designed for low-resource settings, addressing gaps noted in previous reviews: the first efficacy data for bupropion in PLWH [56], combination NRT with intensive counseling and a chronic-care re-treatment model [57, 58], and proactive Quitline referral with tailored nurse-delivered counseling [59]. All three trials used follow-up of at least six months and biochemical verification of abstinence.

Across the 32 studies, participants were broadly similar: most were middle-aged (mean ages 37–55), predominantly male (51%−97%), apart from one trial of female participants only [33], and were moderate to heavy smokers (10–29 cigarettes per day) with medium to high nicotine dependence (FTND 4.0–7.2, where reported). Most studies did not employ masking while eight used some form of blinding: of study participants and staff [28, 48, 55], of outcome evaluators [34, 36, 42], or of both outcome evaluators and investigators [37, 41].

### Smoking cessation interventions

Smoking cessation interventions varied by content, intensity, duration, and mode of delivery. Across studies, interventions were grouped into four clinically relevant categories for synthesis: pharmacological interventions, tailored or intensive behavioral support, peer-navigation or social-support interventions, and system- or process-level interventions. Pharmacological interventions included nicotine replacement therapy, varenicline, and bupropion, either alone or combined with behavioral support. Behavioral interventions ranged from brief advice or standard counseling to intensive, multi-session counseling, culturally adapted programs, web-based support, text messaging, and telephone-based counseling. Sixteen studies tailored the intervention to PLWH-specific physiological, psychological, cultural, or social needs [28, 31–34, 36–39, 41–43, 45, 55, 56, 59], whereas the remaining 16 delivered generic cessation counseling based on published guidelines [29, 30, 35, 40, 44, 46–54, 57, 58]. Detailed synthesis of intervention components by intervention group is provided in S2 Text, and study-level characteristics are provided in S2 Table.

### Risk of bias assessment

Twenty randomized controlled trials were assessed for risk of bias using Cochrane’s risk-of-bias tool RoB 2 (Table 1). Although most studies were assessed as having a “Low” risk of bias, seven studies were rated as having a “Some” risk of bias due to issues such as lack of transparency on the randomization process [35, 39, 44, 55], deviations from the intended interventions [37, 42, 57], and missing outcome data arising from moderate to high loss to follow-up [33, 38, 39]. One factorial trial was rated at high risk of bias because of missing outcome data [41]. Non-randomized studies, assessed with the EPHPP tool, ranged from high to low risk of bias, with most at moderate risk. The ratings were driven mainly by risks of bias arising from participant selection, study design, or blinding (Table 2).

**Table 1:** Risk of bias assessment for randomized studies using Cochrane’s risk-of-bias tool for randomized trials (RoB 2).

First author, year	A	B	C	D	E	Overall bias
***High-income countries***						
Ashare, 2019 [28]	Low	Low	Low	Low	Low	Low
Cioe, 2025 [42]	Low	Some	Low	Low	Low	Some
Edelman, 2026 [44]	Some	Low	Low	Low	Low	Some
Gritz, 2013 [31]	Low	Low	Low	Low	Low	Low
Himelhoch, 2024 [41]	Low	Low	High	Low	Low	High
Humfleet, 2013 [32]	Low	Low	Low	Low	Low	Low
Kim, 2018 [33]	Low	Low	Some	Low	Low	Some
Lloyd-Richardson, 2009 [34]	Low	Low	Low	Low	Low	Low
Mercié, 2018 [48]	Low	Low	Low	Low	Low	Low
Mussulman, 2018 [35]	Some	Some	Low	Low	Low	Some
O’Cleirigh, 2018 [36]	Low	Low	Low	Low	Low	Low
Shuter, 2020 [38]	Low	Some	Some	Low	Low	Some
Shuter, 2022 [37]	Low	Low	Low	Low	Low	Low
Stanton, 2015 [39]	Some	Low	Some	Low	Low	Some
Stanton, 2020 [43]	Low	Low	Low	Low	Low	Low
Tindle, 2022 [55]	Some	Low	Low	Low	Low	Low
***Low-and-middle-income countries***						
Elf, 2025 [57]	Low	Some	Low	Low	Low	Some
Himelhoch, 2024 [56]	Low	Low	Low	Low	Low	Low
Keke, 2026 [58]	Low	Low	Low	Low	Low	Low
Shelley, 2026 [59]	Low	Low	Low	Low	Low	Low

**Note. Low** = Low risk; **Some** = Some concerns; **High** = High risk.; **A** = Randomization process; **B** = Deviations from intended interventions; **C** = Missing outcome data; **D** = Measurement of the outcome; **E** = Selection of the reported result. Elf (2025) and Keke (2026) report the same South African trial and were assessed separately. Two “Himelhoch, 2024” entries are distinct factorial trials conducted in the USA and Kenya, respectively.

**Table 2:** Risk of bias assessment for non-randomized studies using the EPHPP’s Quality Assessment Tool for Quantitative Studies.

First author, year	A	B	C	D	E	F	Final decision
Altobelli, 2026 [51]	Moderate	Moderate	Strong	Weak	Weak	Strong	Weak
Balfour, 2017 [45]	Weak	Moderate	Strong	Moderate	Weak	Weak	Weak
Bui, 2020 [40]	Moderate	Moderate	Moderate	Moderate	Strong	Moderate	Strong
Chew, 2014 [29]	Weak	Moderate	Strong	Moderate	Moderate	Strong	Moderate
Cui, 2012 [46]	Moderate	Moderate	Strong	Weak	Strong	Strong	Moderate
De Socio, 2020 [50]	Moderate	Moderate	Strong	Moderate	Moderate	Weak	Moderate
Edwards, 2022 [47]	Moderate	Moderate	Strong	Weak	Strong	Strong	Moderate
Elzi, 2006 [53]	Weak	Moderate	Strong	Moderate	Weak	Strong	Weak
Fitzgerald, 2016 [30]	Moderate	Moderate	Strong	Moderate	Weak	Weak	Weak
Fuster, 2009 [52]	Weak	Moderate	Strong	Moderate	Strong	Strong	Moderate
Grabovac, 2017 [54]	Weak	Moderate	Strong	Moderate	Strong	Strong	Moderate
Parienti, 2017 [49]	Weak	Moderate	Strong	Moderate	Strong	Moderate	Moderate

**Note. Strong** = Low risk of bias; **Moderate** = Moderate risk of bias; **Weak** = High risk of bias. **A** = Selection bias; **B** = Study design; **C** = Confounders; **D** = Blinding; **E** = Data collection method; **F** = Withdrawals and dropouts.

### Outcome measurement and abstinence definitions

Per-study outcomes, measures, and follow-up periods are presented in Table 3. Outcome definitions and verification methods varied across studies. Most studies used biochemically verified abstinence, while six relied exclusively on self-reported abstinence [29, 30, 47, 50, 51, 53]. Expired carbon monoxide was the most common biochemical verification method, although cut-off thresholds varied across studies, most often ranging from 4 to 10 ppm, with 10 ppm being the most frequently applied threshold [32, 34, 37–39, 41, 43, 45, 48, 55]. Some studies used cotinine testing in serum [33, 35, 46] or urine [57, 58], either instead of or in addition to expired carbon monoxide. Reported abstinence outcomes also varied, including continuous abstinence, 7-day point-prevalence abstinence, and definitive abstinence at the longest follow-up. These differences in outcome definition and verification contributed to heterogeneity across studies and were considered when selecting the strictest available abstinence outcome for synthesis. Detailed outcome definitions, biochemical verification methods, and study-level abstinence rates are provided in S3 Text and S2 Table.

**Table 3:** Smoking cessation outcomes from the included studies.

Author year country study design	Intervention, sample size	Outcome measurement	Intervention outcome
***High-income countries***			
Altobelli et al. (2026) [51]	Brief counseling (standard guideline) (n = 340)	Self-reported definitive abstinence (≥ 6 months and no recurrence at last control visit)	Definitive abstinence at last visit: Standard: 17.4% Soft: 4.1%
	Soft intervention (n = 170)		
Ashare et al. (2019) [28]	Intensive counseling + Pharmacotherapy (n = 89)	Self-reported and expired CO confirmed (cutoff: 8 ppm).	7-day PPA 24 weeks: 14.6% in Varenicline group vs. 10.0% in Placebo group. OR: 1.9 (95%CI: 0.71–5.1) Continuous abstinence 24 weeks: 10.1% in Varenicline group vs. 6.7% in Placebo group.
	Intensive counseling + placebo (n = 90)		
Balfour et al. (2017) [45]	Intensive counseling + NRT (n = 50)	Self-reported and expired CO confirmed (cutoff: 10 ppm).	Continuous abstinence 24 weeks: 28%
Bui et al. (2020) [40]	Brief counseling + Quitline + NRT (n = 214)	Self-reported and expired CO confirmed (cutoff: not reported).	7-day PPA 6 months: 4.2%.
Chew et al. (2014) [29]	Intensive counseling + Pharmacotherapy (n = 123)	Self-reported.	7-day PPA 6 months: 16%
Cioe et al. (2025) [42]	Standard care (n = 34)	Self-reported, expired CO confirmed (cutoff: 5 ppm)	7-day PPA at 24 weeks: Standard care: 6% Peer navigation: 3%
	Peer Navigation (n = 30)		
Cui et al. (2012) [46]	Counseling + Pharmacotherapy (n = 36)	Self-reported and serum cotinine verified.	7-day PPA 24 weeks months: 42% Continuous abstinence 9–24 weeks: 28%
De Socio et al. (2020) [50]	Brief counseling + NRT/Pharmacology (n = 343)	Self-reported.	Continuous abstinence at 6 months: 5.51% in standard intervention vs. 1.65% in soft intervention. Higher reduction in daily cigarettes of standard intervention group.
	Brief counseling + NRT/Pharmacology (n = 218)		
Edelman et al. (2026) [44]	NRT first then Switch to Oral MTUD if failed to quit (N = 86)	7-day PPA at 12 and 24 weeks, confirmed by eCO <= 6 ppm or next closest informant.	Abstinence at 24 weeks for non-responders: NRT (Stage 1) + NRT + CM (Stage 2): 13.7% (4.2%−36.6%) NRT (Stage 1) + Oral MTUD (Stage 2): 10.1% (2.1%−36.9%) NRT + CM (Stage 1) + NRT + CM intensified (Stage 2): 18.0% (4.1%−52.7%) NRT + CM (Stage 1) + Oral MTUN + CM (Stage 2): 11.7% (1.6%−51.8%)
	NRT first then Intensified if failed to quit (n = 90)		
	NRT + CM first then Switch to Oral MTUD if failed to quit (n = 96)		
	NRT + CM first then Intensified if failed to quit (n = 98)		
Edwards et al. (2022) [47]	NRT (n = 29)	Self-reported.	7-day PPA 24 weeks: 28%. Reduction of average nicotine dependence FTND in non-quitters compared to baseline.
Elzi et al. (2006) [53]	Intensive counseling + NRT (n = 34)	Self-report continuous abstinence.	Continuous abstinence for 12 months: 50% vs 15% in control. Continuous abstinence more than 12 months: 38% vs 7% in control (OR: 6.2, 95%CI: 2.8–14.3) Relapse after 6 months (n = 1)
	Nothing (n = 383)		
Fitzgerald et al. (2016) [30]	Brief counseling + Quitline + NRT (n = 50)	Self-reported.	7-day PPA 6 months: 7%
Fuster et al. (2009) [52]	Intensive counseling + Pharmacotherapy (n = 33)	Self-reported and expired CO confirmed (cutoff: 5 ppm).	Continuous abstinence at 6 months: 39.40%. Continuous abstinence at 12 months: 25%.
Grabovac et al. (2017) [54]	Intensive counseling + NRT/Pharmacotherapy (n = 63)	Self-reported and expired CO confirmed (cutoff: 6 ppm).	7-day PPA 8 months: 19% in full program vs. 14.7% in short program. No significant changes in QoL between quitters and non-quitters.
	Brief counseling (n = 102)		
Gritz et al. (2013) [31]	Intensive counseling + NRT (n = 236)	Self-reported and expired CO confirmed (cutoff: 7 ppm)	7-day PPA 12 months: OR: 2.41, 95%CI: 1.01–5.76
	Usual care (n = 238)		
Himelhoch et al. (2024) [41]	PSF varenicline (n = 49)	Self-reported and expired CO confirmed (cutoff: 10 ppm).	7-day PPA at 36 weeks: PSF varenicline: 16.7% PSF placebo: 3.6% SOC varenicline: 13.9% SOC placebo: 10.8%
	PSF placebo (n = 42)		
	SOC varenicline (n = 46)		
	SOC placebo (n = 47)		
Humfleet et al. (2013) [32]	SOC placebo, “IC” (n = 47)	Self-reported and expired CO confirmed (cutoff: 10 ppm)	7-day PPA 24 weeks: 15.09% (IC) vs. 26.67% (CBI) vs. 15.07% (Brief) 7-day PPA 36 weeks: 21.28% (IC) vs. 20.93% (CBI) vs. 18.92% (Brief) 7-day PPA 52 weeks: 20.41% (IC) vs. 25.58% (CBI) vs. 19.72% (Brief) Significant reduction in number of smoking cigarettes overtime: from 17.5 to 8.4 (Brief), from 18.4 to 8.4 (IC), from 17.2 to 10.5 (CBI).
	Intensive counseling + NRT, “CBI” (n = 58)		
	Brief counseling + NRT, “Brief” (n = 82)		
Kim et al. (2018) [33]	Intensive counseling (video call) + NRT (n = 21)	Self-reported and serum cotinine confirmation (cutoff: 0).	Continuous abstinence at 6 months: 38.1% (video-call) vs. 4.8% (voice call). Continuous abstinence for 6 months: 33.3% (video-call) vs. 4.8% (voice call).
	Intensive counseling (voice call) + NRT (n = 21)		
Lloyd-Richardson et al. (2009) [34]	Intensive counseling + NRT (ME) (n = 212)	Self-reported and expired CO confirmed (cutoff: 10 ppm).	7-day PPA 6 months: 9% (ME) vs. 10% (SC)
	Brief counseling + NRT (SC) (n = 232)		
Mercié et al. (2018) [48]	Intensive counseling + Pharmacotherapy (n = 123)	Self-reported and expired CO confirmed (cutoff: 10 ppm).	Continuous abstinence weeks 9–48: 15% (varenicline) vs 6% (placebo); OR: 2.5 (95% CI: 1.0–6.1).
	Intensive counseling + placebo (n = 125)		
Mussulman et al. (2018) [35]	Brief counseling + Quitline (hand-off) + NRT (n = 11)	Self-reported and serum cotinine confirmed (cutoff: 15ng/mL).	7-day PPA 6 months: 45.50% (warm hand-off) vs. 14.30% (fax-referral).
	Brief counseling + Quitline (fax referral) + NRT(n = 14)		
O’Cleirigh et al. (2018) [36]	Intensive counseling + NRT (n = 26)	Self-reported and expired CO confirmed (cutoff: 4 pm)	7-day PPA 6 months: 46% (QUIT) vs. 5% (ETAU).
	Brief counseling + NRT (n = 27)		
Parienti et al. (2017) [49]	Intensive counseling + NRT/Pharmacotherapy (n = 147)	Self-reported and expired CO confirmed (cutoff: Not reported)	7-day PPA 6 months: 40.80%
Shuter et al. (2020) [38]	Intensive counseling + NRT (n = 165)	Self-reported and expired CO confirmed (cutoff: 10 ppm).	7-day PPA 12 months: 10.3% (intensive counseling) vs. 4.2% (brief counseling). OR: 2.61 (95%CI: 1.05–6.47) 7-day PPA late follow-up (over 12 months): 12.70% (intensive counseling) vs 6.60% (brief counseling). OR: 2.06 (95%CI: 0.96–4.41).
	Brief counseling + NRT (n = 166)		
Shuter et al. (2022) [37]	Intensive counseling + NRT (n = 255)	Self-reported and expired CO confirmed (cutoff: 10 ppm).	7-day PPA 6 months: 14.9% (PSFW) vs. 8.8% (AHA), OR: 1.82 (95%CI: 1.04–3.18) No reduction in number of cigarettes among non-quitters.
	Counseling + NRT (n = 251)		
Stanton et al. (2015) [39]	Intensive counseling + NRT (Aurora) (n = 154)	Self-reported and expired CO confirmed (cutoff: 10 ppm).	7-day PPA 6 months: 8% (Aurora) vs. 11% (ESC). No between-group differences. 7-day PPA 12 months: 6% (Aurora) vs. 7% (ESC). No between-group differences. Significant reductions in smoking intensity, number of quit attempts, nicotine dependence FTND in total sample.
	Brief counseling + NRT (ESC) (n = 148)		
Stanton et al. (2020) [43]	Intensive counseling + NRT (n = 216)	Self-reported and expired CO confirmed (cutoff: 10 ppm)	7-day PPA 6 months: 13.0% (PSF) compared to 13.30% (brief counseling). OR: 0.97 (95%CI: 0.56–1.69)
	Brief counseling + NRT (n = 226)		
Tindle et al. (2022) [55]	Brief counseling + Pharmacotherapy (n = 100) – Group 1	Self-reported and expired CO confirmed (cutoff: 10 ppm).	7-day PPA 6 months: 14.1% (group 1) vs. 16.3% (group 2) vs. 20.7% (group 3) vs. 20.2% (group 4). 7-day PPA 12 months: 18.0% (group 1) vs. 18.0% (group 2) vs. 25.0% (group 3) vs. 20.0% (group 4). Reductions in number of daily smoking cigarettes: from 21 (SD:8) to 7.2–8.2 at 12 months.
	Brief counseling + NRT (n = 99) – Group 2		
	Brief counseling + Pharmacotherapy (n = 100) – Group 3		
	Brief counseling + NRT (n = 101) – Group 4		
***Low-and-middle-income countries***			
Elf et al. (2024) [57]	Behavioral counseling (n = 281)	Self-reported, expired CO (cutoff: 7 ppm) and urine cotinine (cutoff: 0.4 µg/mL) confirmed.	7-day PPA at 6 months: BC + c-NRT: 15% BC: 10%
	Behavioral counseling + c-NRT (n = 280)		
Himelhoch et al. (2024) [56]	PSF + bupropion (n = 74)	Self-reported, exhaled CO (cutoff: 7 ppm) confirmed.	7-day PPA at 36 weeks: Bupropion: 31.3% (vs Placebo 13.3%, p < 0.001) PSF: 29.5% (vs SOC 14.9%, p = 0.003) Combined (PSF + Bup): 38.9%.
	PSF + placebo (n = 76)		
	SOC + bupropion (n = 74)		
	SOC + placebo (n = 76)		
Keke et al. (2026) [58]	Repeat Behavioral counseling (n = 196)	Self-reported, expired CO (cutoff: 7 pp) and urine cotinine (cutoff: 0.4 µg/mL) confirmed.	Biochemically confirmed abstinence at 6 months after re-treatment: BC: 8% BC + c-NRT: 11%
	Repeat Behavioral counseling + c-NRT (n = 188)		
Shelley et al. (2026) [59]	Quitline referral (n = 221)	Self-reported, expired CO 7-day PPA (cutoff: 8 ppm) confirmed.	7-day PPA at 6 months (biochemically confirmed): Quitline referral: 13% Counselling+SMS: 18% Counselling+SMS +gum: 18%
	Counselling+SMS (n = 225)		
	Counselling+SMS + gum (n = 226)		

*Abbreviations: ALT: alanine Aminotransferase; ART: anti-retroviral treatment; AST: aspartate aminotransferase; CA: continuous abstinence; CI, confidence interval; CO, carbon monoxide; FTND, Fagerstrom’s Test of Nicotine Dependence; NRT, nicotine replacement therapy; OR, odd ratio; PPA, point prevalence abstinence; PSF, Positive Smoke Free; SD, standard deviation; SOC, standard of care.

### Effectiveness and certainty of evidence

The certainty of evidence, assessed with GRADE, ranged from moderate to very low across the four pooled comparisons (Table 4). Cost and cost-effectiveness outcomes could not be assessed, as no eligible studies reported economic outcomes. Randomized studies contributed to these four pooled comparisons, whereas non-randomized studies were synthesized separately because of differences in design and causal interpretation (S4 Table). Of the 12 non-randomized studies assessed for risk of bias (Table 2), 11 contributed to the certainty-of-evidence synthesis (S4 Table). De Socio et al. (2020) was excluded because it reports an earlier analysis of the same STOPHIV cohort subsequently analyzed with extended follow-up by Altobelli et al. (2026), and retaining both would double-count participants [50, 51]. Evidence from non-randomized studies was rated very low certainty because of study limitations, lack of control groups in several studies, potential confounding, and imprecision. These studies were therefore interpreted narratively and used primarily to contextualize implementation feasibility, longer-term follow-up, and real-world cessation outcomes rather than to support causal conclusions.

**Table 4:** Summary of findings for randomized studies: effectiveness of smoking cessation interventions at six months or longer follow-up.

Comparisons	Assumed risk (placebo/ standard care)	Risk difference with intervention*	Relative effect (95% CI)	Number of participants (studies)	Certainty of evidence (GRADE)
**Pharmacological interventions vs placebo or standard care**	94 per 1,000	80 more per 1,000 (36 more to 121 more)	**RR 1.86** (1.42 to 2.45)	1,471 (5 studies)[28, 41, 48, 56, 57]	⊕⊕⊕⊖ **Moderate**^**a**^
**Tailored or intensive behavioral support vs brief or standard care**	106 per 1,000	38 more per 1,000 (16 more to 71 more)	**RR 1.34** (1.05 to 1.71)	3,606 (10 studies) [32, 34, 36–39, 41, 43, 56, 59]	⊕⊕⊕⊖ **Moderate**^**a**^
**Peer-navigation or social-support interventions vs standard care**	63 per 1,000	42 more per 1,000 (3 fewer to 122 more)	**RR 1.67** (0.95 to 2.94)	570 (2 studies) [37, 42]	⊕⊖⊖⊖ **Very low**^**b**^
**System/process or mode-of-delivery interventions vs standard care**	86 per 1,000	272 more per 1,000 (25 more to 1064 more)	**RR 4.16** (1.29 to 13.37)	67 (2 studies) [33, 35]	⊕⊖⊖⊖ **Very low**^**c**^

**CI:** confidence interval; **RR:** Risk ratio; **PPA:** point-prevalence abstinence.

**GRADE Working Group grades of evidence:**

High quality: We are very confident that the true effect lies close to that of the estimate of the effect.

Moderate quality: We are moderately confident in the effect estimate: The true effect is likely to be close to the estimate of the effect, but there is a possibility that it is substantially different.

Low quality: Our confidence in the effect estimate is limited: The true effect may be substantially different from the estimate of the effect.

Very low quality: We have very little confidence in the effect estimate: The true effect is likely to be substantially different from the estimate of effect.

**Notes**

*The risk in the intervention group (and its 95% confidence interval) is based on the assumed risk in the comparison group and the relative effect of the intervention (and its 95% CI).

^**a**^Downgraded one level for risk of bias: Open-label/unblinded study design poses a risk of performance bias.

^**b**^Downgraded two levels for risk of bias and imprecision: Open-label/unblinded study design poses a risk of performance bias and 95% confidence interval crossed the line of no effect. Widened confidence interval in sensitivity analysis using HKSJ method indicating fragility in the estimate.

^**c**^Downgraded two levels for risk of bias and imprecision: The total number of events does not meet the optimal information size (OIS) criteria. Widened confidence interval in sensitivity analysis using HKSJ method indicating fragility in the estimate.

### Pharmacological interventions

Pharmacological interventions increased smoking abstinence compared with placebo or standard care at six months or longer follow-up, with moderate-certainty evidence (RR 1.86, 95% CI 1.42–2.45; I^2^ = 0%; 1,471 participants; five studies; Table 4 and S1 Fig). The pooled evidence included varenicline, bupropion, and nicotine replacement therapy, usually delivered alongside behavioral support. Subgroup analyses showed statistically significant effects for bupropion and varenicline, while nicotine replacement therapy did not reach statistical significance; these subgroup findings are summarized in S4 Table and shown in S1 Fig. The Kenya trial contributed the first randomized evidence on bupropion for smoking cessation among PLWH and provided one of the few LMIC contributions to the pharmacotherapy evidence base. Sensitivity analyses supported the overall pharmacological effect, although the varenicline and NRT subgroup estimates became less precise under the HKSJ model (S5 Table).

Evidence for additional pharmacotherapy-related subgroup comparisons was limited. Adding combination NRT to repeated behavioral counseling for participants who did not quit initially did not clearly improve abstinence compared with repeated counseling alone [58], while adding financial incentives to standard pharmacotherapy increased quit rates in a single trial [44]. These subgroup findings were rated low certainty because they were based on single studies and are summarized in S4 Table.

### Tailored or intensive behavioral support

Tailored or intensive behavioral support increased smoking abstinence compared with brief or standard care, with moderate-certainty evidence (RR 1.34, 95% CI 1.05–1.71; I^2^ = 32.7%; 3,606 participants; 10 studies; Table 4 and S2 Fig). Interventions in this comparison included PLWH-tailored counseling, culturally adapted behavioral programs, web-based or telephone-based support, and multi-session intensive counseling. This finding should be interpreted as evidence for tailored or intensive intervention packages rather than the isolated effect of counseling alone, because behavioral support was often combined with pharmacotherapy or other implementation components.

The overall finding remained statistically significant in sensitivity analyses, including the HKSJ model (S5 Table). Subgroup analyses by abstinence definition suggested that continuous abstinence outcomes were more robust, whereas point-prevalence abstinence estimates became less precise under the HKSJ adjustment (S5 Table). Publication bias was assessed for this comparison because it included at least 10 studies; visual inspection of the funnel plot and Egger’s test did not indicate clear asymmetry (S3 Fig). LMIC trials from Kenya and Vietnam contributed to this comparison, extending the evidence base beyond the high-income settings that dominated earlier reviews.

### Peer-navigation or social support interventions

Peer-navigation or social-support interventions did not show clear evidence of increased smoking abstinence compared with standard care, and the certainty of evidence was very low (RR 1.67, 95% CI 0.95–2.94; 570 participants; two studies; Table 4 and S4 Fig). Although the point estimate favored intervention, the confidence interval crossed the line of no effect. The evidence was downgraded for study limitations and very serious imprecision, and the HKSJ sensitivity analysis produced a very wide confidence interval, indicating fragility of the pooled estimate (S5 Table). These findings should therefore be interpreted as insufficient evidence rather than evidence that peer-navigation or social-support approaches are ineffective.

### System/process or mode-of-delivery interventions

System/process or mode-of-delivery interventions showed a possible increase in smoking abstinence compared with standard care, but the certainty of evidence was very low (RR 4.16, 95% CI 1.29–13.37; 67 participants; two studies; Table 4 and S5 Fig). The pooled estimate was based on a very small sample and was highly sensitive to model assumptions. In the HKSJ sensitivity analysis, the confidence interval became extremely wide and crossed the line of no effect, indicating substantial uncertainty (S5 Table). These findings suggest that delivery-mode and referral-system changes may be promising, but the current evidence is too uncertain to establish their independent effectiveness.

## Discussion

This systematic review and meta-analysis synthesized evidence on smoking cessation interventions for PLWH and assessed whether cost or cost-effectiveness evidence was available to guide implementation. Pharmacological interventions and tailored or intensive behavioral support increased smoking abstinence at six months or longer follow-up with moderate-certainty evidence, whereas evidence for peer-navigation/social-support and system/process or mode-of-delivery interventions remained very uncertain. Since the 2024 Cochrane review, newly published LMIC trials have added evidence from Kenya and Vietnam, including the first randomized trial of bupropion for smoking cessation among PLWH. However, the overall evidence base remains dominated by studies from high-income settings, and no eligible cost or cost-effectiveness studies were identified. These findings support integration of evidence-based cessation treatment into HIV care while highlighting the need for further implementation and economic evaluations in resource-constrained settings.

The Russell Standard established a common framework for cessation trials, advocating primary outcomes of continuous abstinence at six or twelve months with biochemical verification and intention-to-treat analysis [14]. Despite these criteria, the included studies varied substantially in intervention duration, intensity, delivery mode, and design, and in outcome reporting and biochemical thresholds. This heterogeneity restricts the direct comparability of effect sizes and limits the generalizability of pooled estimates, although it also reflects the range of strategies that have proved feasible for PLWH.

Despite this clinical and methodological heterogeneity, combining data across diverse but related trials is an established method to evaluate the average effect of a broader treatment class, providing the statistical precision necessary to overcome the limited power of isolated studies [15]. Utilizing this approach, our pooled estimate indicates that pharmacological interventions, evaluated as an overall class, increase long-term smoking abstinence compared to placebo or standard care (RR = 1.86). While the included pharmacological interventions have different mechanisms of action, we bundled these comparisons to evaluate the broader clinical utility of integrating any evidence-based pharmacotherapy into standard HIV care. Subgroup analyses support this decision by showing zero statistical heterogeneity (I^2^ = 0%) and consistent treatment effects across the different medication classes.

Our systematic review and meta-analysis complements rather than contradicts the 2024 Cochrane review, as the two syntheses used two different comparison frameworks [12]. Mdege et al. estimated the incremental effect of behavioral support by comparing behavioral support with brief advice while holding pharmacotherapy constant and found no clear benefit. Meanwhile, the present review asked whether tailored or intensive intervention packages, often delivered as multicomponent interventions, outperform brief or standard care. Studies were therefore grouped by intensity and tailoring rather than by isolated component. Heterogeneity for this comparison was modest (I^2^ = 32.7%), and the effect persisted under the HKSJ sensitivity analysis. The estimate therefore reflects the average effect of tailored or intensive intervention packages as a class, rather than the independent effect of any single component.

Several mechanisms may explain why tailored interventions are effective in this population. Such programs commonly address the compounded health risks specific to PLWH, including the cardiovascular and immune consequences of smoking during antiretroviral therapy, and often incorporate strategies to address depression, anxiety, and social isolation, which are prevalent among PLWH and commonly associated with continued smoking [28, 36, 39, 45]. Cultural and social adaptations, including incorporation of population-specific values, HIV peer navigation, and translation of materials for local settings, may further improve engagement and self-efficacy [37, 39, 42, 56]. These adaptations may make cessation support more relevant to the clinical and social context of PLWH, helping to explain why tailored or intensive programs performed better than brief or generic counseling.

A primary distinction of this review is the integration of evidence regarding the mid- to long-term effectiveness of smoking cessation interventions for PLWH in LMICs. While Akanbi et al. [60] reviewed efficacy in general smoker populations, it remained unclear whether these interventions would be similarly effective for PLWH. The 2024 Cochrane review identified only a single trial conducted in an LMIC (South Africa, with unpublished data at the time) and emphasized the critical need for further studies in regions with a high dual burden of HIV and tobacco use [12]. The present review addresses this geographical gap by incorporating three randomized controlled trials from Kenya, South Africa, and Vietnam [56–59]. These trials establish the feasibility of delivering pharmacological and intensive behavioral interventions in resource-constrained settings and provide preliminary efficacy data, though the small number of trials and their mixed results warrant caution.

With evidence regarding clinical feasibility and efficacy in these settings, future research priorities should shift toward implementation science, specifically addressing intervention sustainability and cost-effectiveness. Although formal economic evaluations remain absent from the literature, some trials identified critical operational bottlenecks. Himelhoch et al. noted that while bupropion is an affordable treatment option, the trade-name product was discontinued in Kenya during the trial, identifying supply chain instability as a primary barrier to scalability rather than patient acceptability [56]. Similarly, Elf et al. noted that while NRT is feasible, the out-of-pocket cost remains prohibitive for patients in South Africa without government subsidization [57].

This review has several limitations. First, despite the inclusion of trials from Kenya, South Africa, and Vietnam, the evidence base remains predominantly from the United States. Second, several included trials used open-label designs, introducing potential performance bias, although biochemically verified endpoints mitigated this risk in most studies. Third, the heterogeneity of the evidence required grouping interventions by intensity and tailoring, so the pooled estimates reflect intervention classes rather than isolated components. Finally, data on abstinence beyond 12 months were sparse, and no formal cost-effectiveness analyses for PLWH were identified.

The absence of economic evaluations limits the ability to inform policy in resource-constrained HIV care settings, where cost-effectiveness data are needed to prioritize and scale cessation services. Kahende et al. [61] and a subsequent review of LMIC settings [62] reported that tobacco control can be cost-effective or cost-saving in the general population, but the applicability of these findings to PLWH is uncertain, underscoring the need for economic evaluations tailored to this population.

Despite these limitations, the review has several strengths. It provides a comprehensive synthesis of global evidence through March 2026, addressing gaps identified in prior reviews by incorporating trial data on bupropion and tailored, intensive interventions in LMICs. The use of the GRADE framework provides a transparent assessment of certainty, and the restriction to continuous abstinence and biochemically verified outcomes reduces the risk of overestimating treatment effects.

## Conclusions

This systematic review and meta-analysis provides an updated synthesis of smoking cessation interventions for PLWH through March 2026. We found moderate-certainty evidence that pharmacological interventions and tailored, intensive behavioral support increase long-term smoking abstinence compared with standard care. By incorporating randomized controlled trials from sub-Saharan Africa and Asia, this review begins to address a geographical gap identified in previous literature and provides early evidence for these interventions in LMICs, although that evidence base remains limited. The absence of formal economic evaluations is a key limitation of the global evidence base. Future research should shift from standalone effectiveness trials toward implementation science and cost-effectiveness analyses, addressing operational barriers such as supply chain vulnerability and supporting the sustainable integration of cessation interventions into routine HIV care.

## Supporting information

S1 Checklist The PRISMA 2020 checklist. (DOCX)

S1 Text Supplementary methods. Pooling rules for multi-arm, factorial, and SMART designs; handling of factorial trials contributing to multiple comparisons; and full GRADE domain definitions.
(DOCX)

S2 Text Intervention components by intervention group. (DOCX)

S3 Text Outcome measurement and abstinence definitions. (DOCX)

S1 Table Search strategies and results for PubMed, Cochrane, Scopus, and Web of Science (searched 23 March 2026). (DOCX)

S2 Table Summary of characteristics and results of the included studies. (DOCX)

S3 Table Excluded studies from low- and middle-income countries. (DOCX)

S4 Table Summary of findings for non-randomized studies and subgroup comparisons. (DOCX)

S5 Table Sensitivity analysis results for comparison groups. (DOCX)

S1 Fig Forest plot for pharmacological interventions. (TIF)

S2 Fig Forest plot for tailored or intensive behavioral support versus standard care. (TIF)

S3 Fig Funnel plot for behavioral interventions. (TIF)

S4 Fig Forest plot for peer-navigation or social-support interventions. (TIF)

S5 Fig Forest plot for system/process or mode-of-delivery interventions. (TIF)
