# Supplementary material for: Integrating smoking cessation into HIV care settings: A systematic review and meta-analysis of effectiveness and the evidence gap in cost-effectiveness
Source: PLoS One. 2026 Jul 30;21(7):e0350040. doi: 10.1371/journal.pone.0350040 (PMC13423040; doi:10.1371/journal.pone.0350040)
Supplement: S1 Text — Pooling rules for multi-arm, factorial, and SMART designs; handling of factorial trials contributing to multiple comparisons; and full GRADE domain definitions. (DOCX) [file pone.0350040.s002.docx]

**S1 Text. Supplementary methods: Pooling rules for multi-arm, factorial, and SMART designs; handling of factorial trials contributing to multiple comparisons; and full GRADE domain definitions.**

**1. Handling of complex study designs in the synthesis**

To avoid unit-of-analysis errors and to ensure that each comparison reflected an independent contrast, the following rules were applied when extracting and pooling data from studies with more than two arms or with non-standard designs.

- *Multi-arm trials.* Where a single study included multiple active intervention arms compared against a common control group, the active arms were combined into a single pooled intervention group, which was then compared against the shared control. This prevented control-group participants from being counted more than once.
- *Factorial trials.* For trials using a factorial design, the main effect of the treatment of interest was isolated by pooling all arms that received the active treatment (for example, all arms receiving pharmacotherapy, irrespective of counseling intensity) and comparing them against all arms that received the corresponding control condition (for example, all arms receiving placebo). Because each factor of a factorial trial estimates an independent main effect, such a trial could contribute to more than one comparison, for example, to the pharmacotherapy comparison through its medication factor and to the behavioral comparison through its counseling-intensity factor. In each comparison, only the arms relevant to that factor were pooled, so that no participant contributed to both arms of a single comparison.
- *Trials without a traditional control group.* For trials that did not include a conventional intervention-versus-control structure, such as head-to-head comparative trials and sequential multiple-assignment randomized trials (SMART), arms were not pooled into a single intervention-versus-control contrast. Instead, the relevant arms were extracted to inform specific comparative-efficacy analyses (for example, comparing one pharmacotherapy directly against placebo, with or without enhanced counseling).

**2. Meta-analytic model and estimators**

Effect sizes were pooled as risk ratios using a random-effects model with the generic inverse-variance method, following guidance from the Cochrane Handbook [1]. Between-study variance (τ²) was estimated using the iterative Paule–Mandel estimator [2]. To assess the robustness of the pooled estimates, particularly for comparisons informed by few studies, sensitivity analyses compared the primary Paule–Mandel random-effects model against a fixed-effect model and against the Hartung–Knapp–Sidik–Jonkman (HKSJ) adjustment [3].

**3. GRADE certainty-of-evidence assessment**

The certainty of evidence for each comparison was assessed using the GRADE approach [4]. Randomized trials began at high certainty and non-randomized studies at low certainty. The rating was then downgraded across five domains and where justified, upgraded.

***Downgrading domains***

- **Risk of bias:** limitations in study design or conduct, assessed with the Cochrane RoB 2 tool for randomized trials and the EPHPP tool for non-randomized studies.
- **Inconsistency:** unexplained heterogeneity or variability in results across studies.
- **Indirectness:** differences between the population, intervention, comparator, or outcomes of the included studies and the review question.
- **Imprecision:** wide confidence intervals, small sample sizes, or few events, including confidence intervals that cross the line of no effect or fail to meet the optimal information size.
- **Publication bias:** assessed, for comparisons that included 10 or more studies, by visual inspection of funnel-plot asymmetry and the Egger test.

***Upgrading criteria***

For evidence without serious limitations or plausible residual confounding, certainty could be upgraded by one level when a large effect was observed, defined as a pooled risk ratio greater than 2.0 or less than 0.5.

Certainty was ultimately categorized as high, moderate, low, or very low.
