# Supplementary material for: Integrating smoking cessation into HIV care settings: A systematic review and meta-analysis of effectiveness and the evidence gap in cost-effectiveness
Source: PLoS One. 2026 Jul 30;21(7):e0350040. doi: 10.1371/journal.pone.0350040 (PMC13423040; doi:10.1371/journal.pone.0350040)
Supplement: S2 Text — (DOCX) [file pone.0350040.s003.docx]

**S2 Text. Intervention components by intervention group.**

**Behavioral treatment**

Behavioral treatment typically comprised smoking cessation counseling and reading materials; with one study adding contingency management, offering financial rewards for confirmed abstinence [5]. Counseling intensity varied widely: sessions lasting 5-10 minutes over one or two contacts were classified as "brief" counseling, whereas "intensive" programs delivered 4 to 15 sessions of 30-60 minutes each. This brief-versus-intensive distinction defines the behavioral comparison reported below. Counseling was delivered in person, by telephone (voice or video), or through online platforms, and by a range of clinical and lay personnel; study-level delivery mode and provider type are tabulated in S2 Table.

Building on the main-text classification, the 16 tailored interventions varied in the type of adaptation used, including HIV-specific health education, medication-adherence or symptom-management content, mood or stress-management components, culturally adapted counseling materials, and delivery approaches designed for HIV care settings [6–20]. The 16 generic/non-tailored interventions generally used standard cessation counseling, pharmacotherapy, or system-level support based on existing cessation guidelines. Among the generic programs, several added mechanisms intended to improve effectiveness, such as reinforcing provider adherence to brief counseling at routine visits [21], financial incentives for abstinence [5], and a chronic-care re-treatment model for participants who did not quit initially [22]. Notably, the widely studied “Positively Smoke Free” (PSF) curriculum, an intensive behavioral program developed for PLWH, was delivered across multiple formats in the included studies, including web-based platforms, and was culturally adapted and translated into Swahili for the Kenyan trial [19, 23].

**Pharmacological treatment**

Nicotine replacement therapy (NRT) was provided in various forms, most commonly the patch, with nicotine gum and lozenges also employed, and two studies used combined NRT formulations (e.g., patches plus gum or lozenges) [5, 22, 24]. Two trials used novel forms of NRT, nicotine mouth spray and vaporized nicotine [18, 25]. NRT was typically given for 6 to 12 weeks. In low-resource settings, the South African trial evaluated a 10-week course of combination NRT to manage breakthrough cravings [24], and its extension offered a repeated 10-week course of combination NRT to participants who did not quit initially [22]. In Vietnam, Shelley et al. (2026) provided a 6-week supply of 2 mg nicotine gum provided alongside tailored behavioral in-person counseling in Vietnam [20].

Varenicline was used as pharmacological therapy in seven studies, either as a standalone treatment or in combination with NRT [5, 6, 14, 18, 26–28], and cytisine in one intervention arm [18]. While previous reviews noted the absence of bupropion trials for PLWH, Himelhoch et al. (2024) evaluated sustained bupropion release (150mg twice daily for 12 weeks), providing the first efficacy evidence for this pharmacotherapy among PLWH and in a low-resource setting [19]. Bupropion was also incorporated as a secondary option alongside varenicline in one study [5]. Pharmacotherapy was typically prescribed for 12 weeks.

**Control groups**

Control conditions varied across studies. In ten studies, the control arm received brief counseling (with or without pharmacotherapy) while the treatment arms received intensive counseling [8, 10–14, 16, 19, 23, 29]. Two trials used a placebo control to assess varenicline [6, 27]. One study used “usual care”, described as cessation materials plus information on obtaining NRT from clinics [7], and one non-randomized study used a “no intervention” comparison, contrasting program participants with a cohort of people who smoke and did not enroll [30].

Several trials used active comparators, in which the control arm also received an intervention rather than minimal or no support. This approach narrows the contrast between arms and tends to attenuate the apparent treatment effect. In the South African trial, the control arm received intensive behavioral counseling identical to the intervention arm, isolating the additive benefit of pharmacotherapy [22, 24], while Edelman et al. used an active treatment (NRT alone) as the baseline against the adjunctive benefit of contingency management [5]. Two further trials used comparators more intensive than routine care: a 30–45 minute nurse-led counseling session delivered by online video [15] and proactive referral to Vietnam’s national Quitline, offering up to 10 telephone counseling sessions over a 12-month period [20]. In one trial, the intervention and control groups only differed in the delivery mode (telephone voice-call versus video-call) [9].

Four studies used factorial or adaptive designs without a single fixed control arm [5, 14, 18, 19]: factorial trials crossing cessation medications with placebo [18] or crossing counseling intensity with pharmacotherapy [14, 19], and a sequential multiple-assignment randomized trial (SMART) comparing adaptive treatment strategies [5].
