# Supplementary material for: Integrating smoking cessation into HIV care settings: A systematic review and meta-analysis of effectiveness and the evidence gap in cost-effectiveness
Source: PLoS One. 2026 Jul 30;21(7):e0350040. doi: 10.1371/journal.pone.0350040 (PMC13423040; doi:10.1371/journal.pone.0350040)
Supplement: S3 Text — (DOCX) [file pone.0350040.s004.docx]

**S3 Text. Outcome measurement and abstinence definitions.**

*Abstinence definitions and biochemical verification*

Most studies reported 7-day point-prevalence abstinence (PPA), while eight reported continuous abstinence. Follow-up period was commonly reported at six months or 24 weeks, with the longest follow-up extending to 7.4 years in one study [51]. Of the 32 included studies, 26 used biochemical verification and six relied on self-report alone. Expired carbon monoxide (CO) was the most common method of verification, although CO thresholds varied across studies. Cotinine testing was used less frequently, either in serum or urine.

*Variation in abstinence rates*

Reported abstinence rates varied widely even at comparable follow-up. At six months or 24 weeks, biochemically confirmed 7-day PPA ranged from 3.3% [42] to 46% [36]. This variation may partly reflect differences in outcome definitions, intervention intensity, comparator conditions, follow-up timing, and biochemical verification thresholds. Trials applying stricter CO thresholds (4-5 ppm) reported both the highest and lowest rates, whereas those using more lenient thresholds (6-10 ppm) generally reported abstinence rates ranging from 8 to 27% [28, 32, 34, 37, 39, 43, 44, 54, 55, 57–59]. One trial that analyzed its data at both 6 ppm and 10 ppm found similar rates and effect estimates under either threshold [37].

*Abstinence patterns by intervention and comparator intensity*

Across many comparisons, intervention groups achieved higher abstinence than controls, although effects varied by intervention type and comparator intensity. One of the largest single-study differences was observed for intensive behavioral support combined with pharmacotherapy, achieving a 7-day PPA of 38.9% in an arm receiving intensive counseling and bupropion [56]. Several trials, however, found no significant advantage for more intensive strategies, particularly where the comparator was itself active. For example, in the Vietnamese trial, adding nicotine gum to tailored counseling showed no statistically significant difference in quit rates compared with counseling alone or Quitline referral (18% vs 18% vs 13%) [59].

*Longer-term abstinence and relapse*

Where multiple follow-ups were reported, abstinence typically declined over time, underscoring the difficulty of sustaining long-term cessation [34, 39, 43, 52, 53]. Long-term data were sparse: one non-randomized study reported continuous abstinence of 38% versus 7% beyond 12 months [53], and an extended analysis of the Italian STOPHIV cohort (median follow-up 7.4 years) reported definitive cessation of 17.4% under guideline-adherent brief counseling versus 4.1% under a less intensive intervention [51].

*Secondary outcomes*

Among participants who did not quit, several studies reported reduced daily cigarette consumption, more quit attempts, or lower nicotine dependence [32, 39, 41, 47, 50, 51, 55], and one reported greater reductions in exhaled CO and higher quit motivation with combination NRT [57]. Quality of life and cardiovascular risk were each examined in only one study: smoking was associated with lower baseline quality of life [54], while smoking cessation was associated with reduced 12-month cardiovascular risk [52]. Both rest on a single study and warrant cautious interpretation.
