# Supplementary material for: Integrating smoking cessation into HIV care settings: A systematic review and meta-analysis of effectiveness and the evidence gap in cost-effectiveness
Source: PLoS One. 2026 Jul 30;21(7):e0350040. doi: 10.1371/journal.pone.0350040 (PMC13423040; doi:10.1371/journal.pone.0350040)
Supplement: S1 Table — (DOCX) [file pone.0350040.s005.docx]

**S1 Table. Search strategies and results for PubMed, Cochrane, Scopus, and Web of Science (searched 23 March 2026):**

| **Database** | **Search strategy** | **Search results** |
| --- | --- | --- |
| Pubmed | ("HIV"[MeSH Terms] OR "HIV"[Title/Abstract] OR "Human Immunodeficiency Virus"[Title/Abstract] OR "Acquired Immunodeficiency Syndrome"[MeSH Terms] OR "aids"[Title/Abstract] OR "Acquired Immune Deficiency Syndrome Virus"[Title/Abstract] OR "Acquired Immunodeficiency Syndrome Virus"[Title/Abstract] OR "PLWH"[Title/Abstract]) AND ("smoking cessation"[MeSH Terms] OR "smoking cessation*"[Title/Abstract] OR "tobacco use cessation"[MeSH Terms] OR "tobacco use cessation*"[Title/Abstract] OR "tobacco cessation*"[Title/Abstract] OR "quitting smoking"[Title/Abstract] OR "stop smoking"[Title/Abstract] OR "quit smoking"[Title/Abstract] OR "giving up smoking"[Title/Abstract] OR "Smoking reduction"[Title/Abstract] OR "tobacco reduction"[Title/Abstract]) AND ("abstinen*"[Title/Abstract] OR "relaps*"[All Fields] OR ("cost effective*"[All Fields] OR "cost per QALY gained"[All Fields] OR "Quality-Adjusted Life Years"[MeSH Terms] OR "quality adjusted life year*"[All Fields] OR "life quality"[All Fields] OR "utilit*"[All Fields] OR "daly*"[All Fields] OR "disability adjusted life year*"[All Fields] OR "cost per daly averted"[All Fields] OR "incremental cost effectiveness ratio*"[All Fields] OR "ICER"[All Fields]) OR ("costs and cost analysis"[MeSH Terms] OR ("cost*"[All Fields] AND "analys*"[All Fields]) OR ("econom*"[All Fields] AND "evaluat*"[All Fields]) OR "benefit*"[All Fields] OR "cost minimiz*"[All Fields] OR "total cost"[All Fields] OR "cost allocat*"[All Fields] OR ("cost*"[All Fields] AND ("per quit*"[All Fields] OR "per person"[All Fields] OR "per participa*"[All Fields] OR "per service"[All Fields] OR "per session"[All Fields])) OR "unit cost"[All Fields]) OR (("Risk"[MeSH Terms] OR "risk*"[All Fields] OR "Probability"[MeSH Terms] OR "probabili*"[All Fields]) AND ("comorbidity"[MeSH Terms] OR "comorbid*"[All Fields] OR "Coinfection"[MeSH Terms] OR "coinfect*"[All Fields] OR "cardiovascular diseases"[MeSH Terms] OR "cardiovascular"[All Fields] OR "lung neoplasms"[MeSH Terms] OR "lung cancer"[All Fields] OR "diabetes mellitus, type 2"[MeSH Terms] OR "diabet*"[All Fields] OR "stroke"[MeSH Terms] OR "stroke"[All Fields] OR "pulmonary disease, chronic obstructive"[MeSH Terms] OR "Chronic Obstructive Pulmonary disease"[All Fields] OR "COPD"[All Fields] OR "mortality"[MeSH Terms] OR "mortality"[All Fields])) OR ((("reduc*"[All Fields] OR "decreas*"[All Fields] OR "low"[All Fields] OR "Harm reduction"[MeSH Terms]) AND ("number of cigarettes"[All Fields] OR "nicotine dependen*"[All Fields] OR "Fagerstrom score"[All Fields])) OR (("improve*"[All Fields] OR "chang*"[All Fields] OR "increas*"[All Fields]) AND ("behavio*"[All Fields] OR "read*"[All Fields] OR "self-efficacy"[All Fields])))) | 998 |
| Cochrane | ([mh HIV] OR HIV:ti,ab OR "Human Immunodeficiency Virus":ti,ab OR [mh "Acquired Immunodeficiency Syndrome"] OR aids:ti,ab OR "Acquired Immune Deficiency Syndrome Virus":ti,ab OR "Acquired Immunodeficiency Syndrome Virus":ti,ab OR PLWH:ti,ab) AND ([mh "smoking cessation"] OR ("smoking" NEXT cessation*):ti,ab OR [mh "tobacco use cessation"] OR ("tobacco use" NEXT cessation*):ti,ab OR ("tobacco" NEXT cessation*):ti,ab OR "quitting smoking":ti,ab OR "stop smoking":ti,ab OR "quit smoking":ti,ab OR "giving up smoking":ti,ab OR "Smoking reduction":ti,ab OR "tobacco reduction":ti,ab) AND (abstinen*:ti,ab OR relaps* OR (("cost" NEXT effective*) OR "cost per QALY gained" OR [mh "Quality-Adjusted Life Years"] OR ("quality adjusted life" NEXT year*) OR "life quality" OR utilit* OR daly* OR ("disability adjusted life" NEXT year*) OR "cost per daly averted" OR ("incremental cost effectiveness" NEXT ratio*) OR ICER) OR ([mh "costs and cost analysis"] OR (cost* AND analys*) OR (econom* AND evaluat*) OR benefit* OR ("cost" NEXT minimiz*) OR "total cost" OR ("cost" NEXT allocat*) OR (cost* AND (("per" NEXT quit*) OR "per person" OR ("per" NEXT participa*) OR "per service" OR "per session")) OR "unit cost") OR (([mh Risk] OR risk* OR [mh Probability] OR probabili*) AND ([mh comorbidity] OR comorbid* OR [mh Coinfection] OR coinfect* OR [mh "cardiovascular diseases"] OR cardiovascular OR [mh "lung neoplasms"] OR "lung cancer" OR [mh "diabetes mellitus, type 2"] OR diabet* OR [mh stroke] OR stroke OR [mh "pulmonary disease, chronic obstructive"] OR "Chronic Obstructive Pulmonary disease" OR COPD OR [mh mortality] OR mortality)) OR (((reduc* OR decreas* OR low OR [mh "Harm reduction"]) AND ("number of cigarettes" OR ("nicotine" NEXT dependen*) OR "Fagerstrom score")) OR ((improve* OR chang* OR increas*) AND (behavio* OR read* OR self-efficacy)))) | 349 (Only Trials) |
| Scopus | (INDEXTERMS(HIV) OR TITLE-ABS(HIV) OR TITLE-ABS("Human Immunodeficiency Virus") OR INDEXTERMS("Acquired Immunodeficiency Syndrome") OR TITLE-ABS(aids) OR TITLE-ABS("Acquired Immune Deficiency Syndrome Virus") OR TITLE-ABS("Acquired Immunodeficiency Syndrome Virus") OR TITLE-ABS(PLWH)) AND (INDEXTERMS("smoking cessation") OR TITLE-ABS("smoking cessation*") OR INDEXTERMS("tobacco use cessation") OR TITLE-ABS("tobacco use cessation*") OR TITLE-ABS("tobacco cessation*") OR TITLE-ABS("quitting smoking") OR TITLE-ABS("stop smoking") OR TITLE-ABS("quit smoking") OR TITLE-ABS("giving up smoking") OR TITLE-ABS("Smoking reduction") OR TITLE-ABS("tobacco reduction")) AND (TITLE-ABS(abstinen*) OR ALL(relaps*) OR (ALL("cost effective*") OR ALL("cost per QALY gained") OR INDEXTERMS("Quality-Adjusted Life Years") OR ALL("quality adjusted life year*") OR ALL("life quality") OR ALL(utilit*) OR ALL(daly*) OR ALL("disability adjusted life year*") OR ALL("cost per daly averted") OR ALL("incremental cost effectiveness ratio*") OR ALL(ICER)) OR (INDEXTERMS("costs and cost analysis") OR (ALL(cost*) AND ALL(analys*)) OR (ALL(econom*) AND ALL(evaluat*)) OR ALL(benefit*) OR ALL("cost minimiz*") OR ALL("total cost") OR ALL("cost allocat*") OR (ALL(cost*) AND (ALL("per quit*") OR ALL("per person") OR ALL("per participa*") OR ALL("per service") OR ALL("per session"))) OR ALL("unit cost")) OR ((INDEXTERMS(Risk) OR ALL(risk*) OR INDEXTERMS(Probability) OR ALL(probabili*)) AND (INDEXTERMS(comorbidity) OR ALL(comorbid*) OR INDEXTERMS(Coinfection) OR ALL(coinfect*) OR INDEXTERMS("cardiovascular diseases") OR ALL(cardiovascular) OR INDEXTERMS("lung neoplasms") OR ALL("lung cancer") OR INDEXTERMS("diabetes mellitus, type 2") OR ALL(diabet*) OR INDEXTERMS(stroke) OR ALL(stroke) OR INDEXTERMS("pulmonary disease, chronic obstructive") OR ALL("Chronic Obstructive Pulmonary disease") OR ALL(COPD) OR INDEXTERMS(mortality) OR ALL(mortality))) OR (((ALL(reduc*) OR ALL(decreas*) OR ALL(low) OR INDEXTERMS("Harm reduction")) AND (ALL("number of cigarettes") OR ALL("nicotine dependen*") OR ALL("Fagerstrom score"))) OR ((ALL(improve*) OR ALL(chang*) OR ALL(increas*)) AND (ALL(behavio*) OR ALL(read*) OR ALL(self-efficacy))))) | 1,798 |
| Web of Science | (ALL=HIV OR (TI=HIV OR AB=HIV) OR (TI="Human Immunodeficiency Virus" OR AB="Human Immunodeficiency Virus") OR ALL="Acquired Immunodeficiency Syndrome" OR (TI=aids OR AB=aids) OR (TI="Acquired Immune Deficiency Syndrome Virus" OR AB="Acquired Immune Deficiency Syndrome Virus") OR (TI="Acquired Immunodeficiency Syndrome Virus" OR AB="Acquired Immunodeficiency Syndrome Virus") OR (TI=PLWH OR AB=PLWH)) AND (ALL="smoking cessation" OR (TI="smoking cessation*" OR AB="smoking cessation*") OR ALL="tobacco use cessation" OR (TI="tobacco use cessation*" OR AB="tobacco use cessation*") OR (TI="tobacco cessation*" OR AB="tobacco cessation*") OR (TI="quitting smoking" OR AB="quitting smoking") OR (TI="stop smoking" OR AB="stop smoking") OR (TI="quit smoking" OR AB="quit smoking") OR (TI="giving up smoking" OR AB="giving up smoking") OR (TI="Smoking reduction" OR AB="Smoking reduction") OR (TI="tobacco reduction" OR AB="tobacco reduction")) AND ((TI=abstinen* OR AB=abstinen*) OR ALL=relaps* OR (ALL="cost effective*" OR ALL="cost per QALY gained" OR ALL="Quality-Adjusted Life Years" OR ALL="quality adjusted life year*" OR ALL="life quality" OR ALL=utilit* OR ALL=daly* OR ALL="disability adjusted life year*" OR ALL="cost per daly averted" OR ALL="incremental cost effectiveness ratio*" OR ALL=ICER) OR (ALL="costs and cost analysis" OR (ALL=cost* AND ALL=analys*) OR (ALL=econom* AND ALL=evaluat*) OR ALL=benefit* OR ALL="cost minimiz*" OR ALL="total cost" OR ALL="cost allocat*" OR (ALL=cost* AND (ALL="per quit*" OR ALL="per person" OR ALL="per participa*" OR ALL="per service" OR ALL="per session")) OR ALL="unit cost") OR ((ALL=Risk OR ALL=risk* OR ALL=Probability OR ALL=probabili*) AND (ALL=comorbidity OR ALL=comorbid* OR ALL=Coinfection OR ALL=coinfect* OR ALL="cardiovascular diseases" OR ALL=cardiovascular OR ALL="lung neoplasms" OR ALL="lung cancer" OR ALL="diabetes mellitus, type 2" OR ALL=diabet* OR ALL=stroke OR ALL=stroke OR ALL="pulmonary disease, chronic obstructive" OR ALL="Chronic Obstructive Pulmonary disease" OR ALL=COPD OR ALL=mortality OR ALL=mortality)) OR (((ALL=reduc* OR ALL=decreas* OR ALL=low OR ALL="Harm reduction") AND (ALL="number of cigarettes" OR ALL="nicotine dependen*" OR ALL="Fagerstrom score")) OR ((ALL=improve* OR ALL=chang* OR ALL=increas*) AND (ALL=behavio* OR ALL=read* OR ALL=self-efficacy)))) | 2,153 |
