# Supplementary material for: Integrating smoking cessation into HIV care settings: A systematic review and meta-analysis of effectiveness and the evidence gap in cost-effectiveness
Source: PLoS One. 2026 Jul 30;21(7):e0350040. doi: 10.1371/journal.pone.0350040 (PMC13423040; doi:10.1371/journal.pone.0350040)
Supplement: S2 Table — (DOCX) [file pone.0350040.s006.docx]

**S2 Table. Summary of characteristics and results of the included studies.**

| **Author**  **Year**  **Country**  **Study design** | **Eligible criteria** | **Service group, sample size** | **Description** | **Counseling**  **person** | **Participants characteristics** | **Outcome measures and abstinence rates** | **Findings and limitations** |
| --- | --- | --- | --- | --- | --- | --- | --- |
| ***High-income countries*** | | | | | | | |
| Altobelli et al. [1]  2026  Italy  Multicenter cohort prospective study, single-group, no blinding. | *Inclusion:* PLWH, current smokers at baseline, attended scheduled or unscheduled visits at participating centers, enrolled in the STOPHIV project.  *Exclusion:* Participants enrolled in 2/10 centers which did not adhere to the prolonged follow-up. | Brief counseling (standard guideline)  (n=340) | Brief counseling at baseline, repeated every 6 months for 2 years, at least one repetition every year.  (≥3 brief counseling sessions over 2 years) | Trained physicians following the EACS guidelines | Mean age, SD: 47.2, 9.9  77.6% Male  92.9% Caucasian  Median FTND, IQR: 4 (1-5) | Self-reported definitive abstinence (≥ 6 months and no recurrence at last control visit)  Definitive abstinence at last visit:  Standard: 17.4%  Soft: 4.1% | Adherence to brief intervention is a critical factor for effectiveness. Standard intervention was significantly more effective. Smoking cessation was associated with no detectable HIV RNA.  Limitations: Non-randomized observational design (assigned afterward based on clinician adherence), self-reported outcomes without biochemical verification, potential unmeasured confounders |
|  |  | Soft intervention (n=170) | Brief counseling at baseline, but had <3 sessions over 2 years. |  | Mean age, SD: 46.8, 9.1  71.8% Male  95.9% Caucasian  Median FTND, IQR: 5 (3-8) |  |  |
| Ashare et al. [2]  2019  USA  Placebo-controlled, randomized trial, double-blinded. | *Inclusion:* Age >18, confirmed HIV; ART treatment; <1000 copies/ml; CD4+ counts >200 cells/mm3; daily smoking; ALT and AST <2 times upper limit of normal, and creatinine clearance >50 mL/min.  *Exclusion:* Self-reported psychological history (psychosis, a suicide attempt); Pregnancy (current/planning); ongoing smoking cessation medications; alcohol/substance abuse (uncontrolled/untreated); uncontrolled hypertension (systolic>160 or diastolic>100). | Intensive counseling + Pharmacotherapy (n=89) | Six counseling sessions in-person or by telephone.  Titrated varenicline dose over 12 weeks. | Trained counselors, supervised by psychologist | Mean age, SD: 48.6 (9.9)  81.5% African American;  68.2% male;  Mean cigarettes per day, SD: 11.5 (7.9);  Mean breath CO, SD: 14.1 ppm (9.2). | Self-reported and Expired CO confirmed (cutoff: 8ppm).  7-day PPA 24 weeks: 14.6% in Varenicline group vs. 10.0% in Placebo group. OR: 1.9 (95%CI: 0.71-5.1)  CA 24 weeks: 10.1% in Varenicline group vs. 6.7% in Placebo group. | Varenicline is safe and its efficacy is significant compared to placebo at all follow-ups, however, decreased over time. Abstinence rates at Week 24 is not significant different between two groups.  Limitations: low representative of general HIV smokers due to strict eligible criteria; Low observed adherence. |
|  |  | Intensive counseling + placebo (n=90) | Six counseling sessions in-person or by telephone.  Similar packaging placebo. |  |  |  |  |
| Balfour et al. [3]  2017  Canada  Single-group, longitudinal, non-randomized, open-label quasi-experimental study | *Inclusion:* Age >18, confirmed HIV; ART treatment; daily smoking (>10 cigarettes per day); willing to quit within 30 days; Able to read and converse in English  *Exclusion:* Pregnancy (current/planning), breastfeeding; NRT contraindications. | Intensive counseling + NRT (n=50) | Five in-person counselling sessions (45-60 minutes).  Nicotine patches for 10 weeks (varies by number of smoking cigarettes) | Fellow psychologists | Mean age, SD: 45.1 (8.7);  95.9% Caucasian;  88.0% male;  Mean cigarettes per day, SD: 18.17 (6.0)  Mean breath CO, SD: 19.6 (8.2)  Mean FTND, SD: 5.9 (2.5) | Self-reported and Expired CO confirmed (cutoff: 10ppm).  CA 24 weeks: 28% | Inclusion of targeted cognitive-behavioral intervention for depressive symptoms may have improved the abstinence rates. The intervention reduced nicotine dependence FTND scores in those who were still smoking and reduced depressive symptoms.    Limitations: Low external validity, small sample size, participants characteristics (mainly Caucasian men, gay), no control group, no NRT adherence measured. |
| Bui et al. [4]  2020  USA  Prospective cohort study, single-group, no blinding. | *Inclusion*: Age > 18; current smoking; visited study clinic(s) during implementation period. | Brief counseling + Quitline + NRT (n=214) | One brief counseling session and referred to the Texas Quitline. NRT based on individual needs. | Trained nurses and Quitline counsellors | Not reported. | Self-reported and expired CO confirmed (cutoff: not reported).  7-day PPA 6 months: 4.2%. | No significant differences in Quitline enrollment and abstinence rates between HIV and non-HIV patients. NRT use was not statistically associated with higher self-report 7-day PPA among PLWH.  Limitations: Small sample size, lack of individual-level data for further analysis. |
| Chew et al. [5]  2014  USA  Prospective cohort study, no blinding. | *Inclusion*:  Confirmed HIV, current smoker, visited clinic, interested in quitting. | Intensive counseling + Pharmacotherapy (n=123) | At least one individualized face-to-face counseling session (about 1 hour);  Smoking cessation medications prescribed at the initial counseling or follow-up visits. | Trained physician, case manager, peer navigator, mental health counsellor | Mean age, SD: 50.0 (9.1);  85.4% African American;  52.8% male;  Mean cigarettes per day: 11.0;  Mean FTND: 4.6. | Self-reported.  7-day PPA 6 months: 16% | Six months PPA rate was 16% among participants. Some factors associated with non-abstinence: history/current substance use (cocaine, heroin). Factors associated with abstinence: Low nicotine dependence FTND, high readiness to quit. Low adherence observed.  Limitations: No control group, non-randomized, referral bias, small sample size, no biochemical confirmation. |
| Cioe et al. [6]  2025  USA  Randomized controlled trial, open-label, outcome evaluators blinded | *Inclusion:* Diagnosed with HIV; Age >= 18; Smoker >= 5 cigarettes per day for at least a year; positive saliva cotinine test.  *Exclusion:* Concurrent use of | Standard care (n= 34) | One counseling session + Referral to Quitline + Medications (if desired) | Study nurse, peer navigators | Mean age, SD: 54.5 (10.9)  59% Male  60% Caucasian  23% Hispanic  Mean cigarettes per day, SD: 16 (9.1)  Mean FTND’s score, SD: 5.8 (2.1) | Expired CO (cutoff: 5ppm)  7-day PPA at 24 weeks, biochemically confirmed:  SC: 5.9%  PNSS-S: 3.3% | Smoking abstinence rates verified by expired CO among SC and PPNS-S groups were not statistically significant, however, high retention (91% at 24 weeks), high engagement (mean 8.9 of 12 calls completed). Treatment satisfaction was significantly higher in the PNSS-S group (p=.001). Social Support: Positive social support for quitting increased significantly in the PNSS-S group (p=.01) but not in the SC group.  Limitations: Small sample size (pilot study) limited statistical power. Single site recruitment limits generalizability. COVID-19 impact: Transition to remote procedures (Zoom) may have altered engagement and made biochemical verification difficult (only 3 of 8 self-reported quitters had bio-verification). Hawthorne Effect: Intense nurse counseling in the control arm may have inflated control quit rates. |
|  |  | Peer Navigation (n=30) | Standard care + 12 weekly phone counseling sessions with peer navigator |  |  |  |  |
| Cui et al. [7]  2012  Canada  Multicenter, open-label, quasi-experimental study | *Inclusion:* Age 19–64; daily smoking; attempted to quit; abstinent less than 3 months last year; weight 45–125 kg, ART treatment or CD4+ count more than 350 cells/mm3  *Exclusion:* Pregnant or nursing women; allergic to varenicline; cancer or transplant; neural, psychological, cardiovascular, gastrointestinal issues (untreated/on medications); currently on NRT. | Counseling + Pharmacotherapy (n=36) | Titrated varenicline for 12 weeks; Reading materials;  Counseling by a physician or a trained counselor. | Trained physician or counsellor | Mean age, SD: 46 (8)  92% Caucasian;  97% male;  Mean cigarettes per day, SD: 19 (10);  Mean FTND, SD: 5.2 (2.2) | Self-reported and serum cotinine-verified.  7-day PPA 24 weeks: 42%  CA 9-24 weeks: 28% | Varenicline is generally safe to use among HIV-positive smokers. CA and PPA rates at 24 weeks were comparable to pooled results from literature.  Limitations: small sample size, non-randomized open-label study design. |
| De Socio et al. [8]  2020  Italy  Multicentre, cohort prospective study, single-group, no blinding. | *Inclusion:* Age >= 18; confirmed HIV, visited clinics[8]. | Brief counseling + NRT/Pharmacology (n=343) | Standard 5As: At least 3 in-person counseling sessions. | Trained physicians | Mean age, SD: 47.2 (10.1);  92.7% Caucasian;  77.8% male;  Median FTND, IQR: 4 (1-5) | Self-report  CA 6 months: 10.2% in standard intervention vs. 2.8% in soft intervention.  Higher reduction in daily cigarettes of standard intervention group. | Higher adherence (more counseling sessions) resulted in higher continuous abstinence rates for at least 6 months, significant reduction in cigarettes smoking. Counseling had low effect on precontemplation stage of readiness to quit and high FTND score.  Limitations: Non-randomized, no control group. |
|  |  | Brief counseling + NRT/Pharmacology (n=218) | Soft 5As: Less than 2 in-person counseling sessions. | Trained physicians | Mean age, SD: 46.6 (9.3);  96.3% Caucasian;  72.0% male;  Median FTND, IQR: 5 (2-7) |  |  |
| Edelman et al. [9]  2026  USA  Sequential multiple assignment randomized clinical trial, open-label | *Inclusion:* Age >= 18; confirmed HIV status, receiving care at one of the participating sites, current cigarette smokers, smoked ≥ 100 cigarettes lifetime and ≥5 cigarettes daily, provide consent.  *Exclusion*: Not smoking cigarettes, current NRT, bupropion or varenicline use, Pregnancy (current/planning), serious medical or psychological conditions, unable to provide 1 collateral family member or friend, living out of state, unable to read or understand English | NRT (Stage 1 + 2) | Stage 1:  NRT: Dual NRT with brief counseling  NRT+CM: Dual NRT + rewards for confirmed abstinence (up to $350)  Stage 2:  Responders (abstinent at 12 weeks) continued the assignment, non-responders re-randomized into:  Oral MTUD: Varenicline or bupropion.  Intensified CM: More rewards for abstinence (up to $850). | Residency-trained clinical pharmacists. | Mean age, SD: 55.7 (10.8)  73.0% Black/African American  56.4% Male  Median cigarettes per day, IQR: 10 (8-20)  Median breath CO, IQR: 10 (6-16) | Mean CPD and 7-day PPA at 12 and 24 weeks, confirmed by eCO <= 6 ppm or next closest informant.  Abstinence at 24 weeks for non-responders:  NRT (Stage 1) + NRT + CM (Stage 2): 13.7% (4.2%-36.6%)  NRT (Stage 1) + Oral MTUD (Stage 2): 10.1% (2.1%-36.9%)  NRT + CM (Stage 1) + NRT + CM intensified (Stage 2): 18.0% (4.1%-52.7%)  NRT + CM (Stage 1) + Oral MTUN + CM (Stage 2): 11.7% (1.6%-51.8%)  Reduction in cigarettes per day:  NRT (Stage 1) + NRT + CM (Stage 2): 3 (1.3 – 4.8)  NRT (Stage 1) + Oral MTUD (Stage 2): 6.8 (5.1-8.5)  NRT + CM (Stage 1) + NRT + CM intensified (Stage 2): 5.1 (3.2-6.9)  NRT + CM (Stage 1) + Oral MTUN + CM (Stage 2): 4.8 (2.9-6.7) | Adding Contingency Management (CM) to Nicotine Replacement Therapy (NRT) initially improved 12-week abstinence, but not cigarette reduction. Ultimately, the optimal adaptive strategy depends on the clinical goal: to maximize complete abstinence, patients should start with NRT plus CM and intensify CM if they do not respond; conversely, to reduce cigarettes per day, patients should start with NRT alone and add CM if they do not respond.  *Limitations*: COVID-19 impacted recruitment/sample size, low oral MTUD prescribing, participants received prescriptions rather than medications (access issues), next closest informant used for missing expired CO, urban northeast US clinics may limit generalizability |
|  |  | NRT (Stage 1) + NRT + CM (Stage 2) |  |  |  |  |  |
|  |  | NRT (Stage 1) + Oral MTUD (Stage 2) |  |  |  |  |  |
|  |  | NRT + CM (Stage 1+2) |  |  | Mean age, SD: 55.7 (10.6)  72.2% Black/African American  54.7% Male  Median cigarettes per day, IQR: 10 (7-17.5)  Median breath CO, IQR: 10 (6-61) |  |  |
|  |  | NRT + CM (Stage 1) + NRT + CM intensified (Stage 2) |  |  |  |  |  |
|  |  | NRT + CM (Stage 1) + Oral MTUN + CM (Stage 2) |  |  |  |  |  |
| Edwards et al. [10]  2022  New Zealand  Mixed-methods, quasi-experimental study, no blinding. | *Inclusion*: Age >= 18; confirmed HIV; daily smoking; willing to quit.  *Exclusion*: Enrolled in another program; pregnant (current/planning); nursing; experience chest pain; had cardiovascular event or procedure in the last month; active oxygen therapy. | NRT (29) | 12 weeks of using vaporized (Vape) NRT. | None | Mean age, SD: 42 (8)  96% Male  Nicotine dependence (FTND): 8+ (13.8%), 5-7 (34.5%), 3-4 (37.9%), 1-2 (13.8%) | Self-reported.  7-day PPA 24 weeks: 28%.  Reduction of average nicotine dependence FTND in non-quitters compared to baseline. | Nicotine vaporized products are potentially safe, feasible and improve short-term and medium-term abstinence. |
| Elzi et al. [11]  2006  Switzerland  Single-group, longitudinal, non-randomized, open-label quasi-experimental study | *Inclusion*: Age >16, confirmed HIV, interested to quit. | Intensive counseling + NRT (n=34) | Smoking cessation program (SCP): Thirteen counseling sessions delivered by trained nurses (30 minutes).  Nicotine replacement therapy, based on individual needs. | Trained nurses | Mean age, range: 43 (23-63)  97% Caucasian;  82% male;  Mean cigarettes per day, range: 28 (5-69) | Self-report continuous abstinence.  CA more than 12 months: 38% vs 7% in control (OR: 6.2, 95%CI: 2.8-14.3)  Relapse after 6 months (n=1) | Significant continuous abstinence at 12 months among SCP participants compared to control group. Feasibility of SCP delivered by nurses.  Limitations: small sample size, non-randomized, high quit motivation participants. |
|  |  | Nothing (n=383) | No active treatment. | None | Mean age, range: 40 (18-76)  86% Caucasian;  67% male;  Mean cigarettes per day, range: 21 (1-80) |  |  |
| Fitzgerald et al. [12]  2016  USA  Single-group, retrospective cohort study, no blinding. | *Inclusion:* Admitted HIV patients, available electrical health information. | Brief counseling + Quitline + NRT (n=50) | Inpatient bedside counseling, referral to the state tobacco quitline; smoking cessation medication(s). | Counsellor | Mean age, SD: 44.2 (10.4)  95.3% non-Hispanic  74.4% male  Mean cigarettes per day, SD: 13.4 (10.3) | Self-reported.  7-day PPA 6 months: 7% | Few HIV-positive smokers were offered smoking cessation treatment. High proportion of treated patients made quit attempt. Hospitalization offers an opportunity to offer smoking cessation treatment.  Limitations: Low external validity due to small sample size, few received treatment, few were available for follow-up, no control group. bedside service and one hospital setting. |
| Fuster et al. [13]  2009  Spain  Longitudinal descriptive study, single-group, no blinding. | *Inclusion:* Age >18, confirmed HIV, willing to quit, non-drug users, former drug users on treatment; active drug user with less than once a week consumption.  *Exclusion*: Active drug users with frequent consumption. | Intensive counseling + Pharmacotherapy (n=33) | Six group discussion sessions (1 hour)  Pharmacological treatment based on individual needs. | Group discussion | Mean age, SD: 46.0 (6.9)  75.8% male;  Mean cigarettes per day, SD: 21.0 (4.4)  Mean FTND, SD: 5.5 (3.0) | Self-reported and Expired CO confirmed (cutoff: 5ppm).  CA 6 months: 30.3%.  CA 12 months: 25%. | Successful quit patients had lower baseline BMI, anxiety scores, and higher quit motivation. Smoking cessation programs be implemented due to high smoking prevalence among PLWH.  Limitations: Non-randomized, no control, small sample size. |
| Grabovac et al. [14]  2017  Austria and Germany  Multicenter, non-randomized study, no blinding. | *Inclusion:* Age > 18, confirmed HIV. | Intensive counseling + NRT/Pharmacotherapy (n=63) | Full program: One brief counseling session and five intensive counseling sessions (30 minutes).  NRT and pharmacotherapy based on individual needs. | Trained physicians | Mean age: 43.2  88.2% male;  Number cigarettes per day: 0-10 (24%), 11-20 (37.1%), 21-30 (22.2%), >30 (13.1%), missing (3.6%).  Mean breath CO: 16.9 ppm | Self-reported and Expired CO confirmed (cutoff: 6ppm).  7-day PPA 8 months: 19% in full program vs. 14.7% in short program.  No significant changes in QoL between quitters and non-quitters. | Smokers reported lower physical and psychological domain of baseline QoL. QoL reduced as number of smoking cigarettes increased. No significant overtime changes in QoL among smokers, but lower QoL in quitters (not statistically significant).  Limitations: Short study duration, small sample size. |
|  |  | Brief counseling (n=102) | Short program: One brief counseling (5 minutes) | Trained physicians |  |  |  |
| Gritz et al. [15]  2013  USA  Randomized control trial, no blinding. | *Inclusion:* Age >= 18, confirmed HIV, current smoker, expired CO >= 7ppm, willing to quit, speak English or Spanish.  *Exclusion*: current in smoking cessation program and/or deemed ineligible by physicians. | Intensive counseling + NRT (n=236) | Eleven phone-based counseling and access to supportive hotline.  NRT obtained from the clinic and HIV-tailored reading materials. | Trained counselors, supervised by psychologist | Mean age, SD: 43.9 (8.3)  74.6% African American  71.2% Male  Mean cigarettes per day, SD: 18.6 (11.3)  Mean FTND, SD: 5.7 (2.3) | Self-reported and Expired CO confirmed (cutoff: 7ppm)  Overall 7-day PPA through 12 months: OR: 2.41, 95%CI: 1.01-5.76 | Low abstinence rates in both groups, potentially due to low treatment adherence, high FTND scores, high depressive symptoms, low physical health function.  Limitations: Single HIV clinic, no HIV stage stratification, potential misclassification by expired CO due to marijuana use. |
|  |  | Usual care (n=238) | NRT obtained from the clinic and HIV-tailored reading materials. | Trained counselors, supervised by psychologist | Mean age, SD: 45.7 (7.8)  77.7% African American  68.9% Male  Mean cigarettes per day, SD: 19.7 (11.8)  Mean FTND, SD: 5.8 (2.3) |  |  |
| Himelhoch et al. [16]  2024  USA  Factorial design (2x2), randomized, outcome evaluators and investigators blinded. | *Inclusion:* Age >= 18; confirmed HIV; current cigarette smoker; willing to quit; speak English.  *Exclusion:* recently use varenicline; allergic or hypersensitive to varenicline; pregnancy or nursing; renal impairment; unstable cardiovascular disease; substance use disorder; risk of dementia; currently use medications that would interfere with bupropion; medically unstable. | PSF varenicline (n=49) | 8 sessions intensive counselling + 12 weeks Varenicline | Trained provider | Mean age, SD: 52.8 (9.6)  89.7% Black  62.8% Male  Mean cigarettes per day, SD: 11.3 (6.2)  Mean FTND’s score, SD: 4.5 (1.9) | Self-reported and Expired CO confirmed (cutoff: 10ppm).  7-day PPA at 36 weeks:  PSF varenicline: 16.7%  PSF placebo: 3.6%  SOC varenicline: 13.9%  SOC placebo: 10.8% | Noticeable reduction in number of cigarettes per day at all intervention arms and at all follow-ups.  PSF did not have better cessation outcomes compared to brief counseling at 36 weeks.  None of the odd ratios were statistically significant, potentially due to the small size.  Varenicline was not found to be associated with any adverse effects.  Limitations: failed to meet recruitment goal; self-reported adherence may introduce bias toward null hypothesis; urban settings which limited generalizability. |
|  |  | PSF placebo (n=42) | 8 sessions intensive counselling + 12 weeks Placebo | Trained provider |  |  |  |
|  |  | SOC varenicline (n=46) | Quit smoking brochure + Brief counselling + 12 weeks Varenicline | Trained study staff |  |  |  |
|  |  | SOC placebo (n=47) | Quit smoking brochure + Brief counselling + 12 weeks Placebo | Trained study staff |  |  |  |
| Humfleet et al. [17]  2013  USA  Randomized control trial, no blinding. | *Inclusion:* Age >= 18, current smokers, registered patients at clinic.  *Exclusion*: current in smoking cessation program; experiencing cognitive impairment or dementia. | SOC placebo (n=47) | Quit smoking brochure + Brief counselling + 12 weeks Placebo | Trained study staff | Mean age, SD: 45 (8)  52.7% Caucasian  81.7% Male  Mean cigarettes per day, SD: 19.8 (10.5)  Mean FTND, SD: 4.9 (2.4) | Self-reported and Expired CO confirmed (cutoff: 10 ppm)  7-day PPA 24 weeks: 15.09% (IC) vs. 26.67% (CBI) vs. 15.07% (Brief)  7-day PPA 36 weeks: 21.28% (IC) vs. 20.93% (CBI) vs. 18.92% (Brief)  7-day PPA 52 weeks: 20.41% (IC) vs. 25.58% (CBI) vs. 19.72% (Brief)  Significant reduction in number of smoking cigarettes overtime: from 17.5 to .8.4 (Brief), from 18.4 to 8.4 (IC), from 17.2 to 10.5 (CBI). | CBI program obtained higher abstinence rates, but no significant differences observed among interventions.  Limitations: No HIV-related data, low external validity, not designed to differentiate efficacy among programs. |
|  |  | Intensive counseling + NRT (n=58) | Computer-based intervention (CBI): Interactive, computer-based website, five sessions (30-45 mins).  Ten weeks of NRT | Research staff |  |  |  |
|  |  | Brief counseling + NRT (n=82) | Brief: One brief counseling, reading materials.  Ten weeks of NRT. | Research staff |  |  |  |
| Kim et al. [18]  2018  USA  Randomized control trial, no blinding. | *Inclusion:* Women; age 18-75; speak English; confirmed HIV, CD4 cell count and viral load; daily smoking; access to video calling, via smartphone; willingness to quit 4, birth control during the study period  *Exclusion:* not speak English; pregnant or lactating; an active skin disease; serious alcohol use; history of serious mental illness; illegal substance use. | Intensive counseling + NRT (n=21) | Eight video-call counseling sessions (30 minutes).  Eight weeks of nicotine patches. | Therapist | Mean age, SD: 51.1 (7.7)  73.8% African American  100% Female  Mean cigarettes per day, SD: 14.2 (6.7)  Mean FTND, SD: 5.6 (1.8) | Self-reported and serum cotinine confirmation (cutoff: 0).  CA at 6 months: 38.1% (video-call) vs. 4.8% (voice call). | Video-call counseling showed a large effect on smoking cessation among participants compared to voice-call. Video-call arm participants were also more likely to maintain abstinence over 6 months. No difference in feasibility and acceptability of two delivery modes. Significantly higher rate of retention in video-call than in voice-call.  Limitations: Low external validity due to small size, low representativeness and high quit motivation sample. |
|  |  | Intensive counseling + NRT (n=21) | Eight phone-based counseling sessions (30 minutes).  Eight weeks of nicotine patches. | Therapist |  |  |  |
| Lloyd-Richardson et al. [19]  2009  USA  Randomized control trial, outcomes evaluator blinded. | *Inclusion*: Age > 18; confirmed HIV; daily smoking; available in the next six months.  *Exclusion*: NRT contraindications; current using smoking cessation; pregnant or nursing | Intensive counseling + NRT (n=212) | Motivationally-enhanced (ME): Four intensive counseling sessions (30 minutes).  Eight weeks of NRT. | Health Educator | Mean age, SD: 42.1 (7.7)  51.8% European American  62.3% Male  Mean cigarettes per day, SD: 18.3 (9.8)  Mean FTND, SD: 5.9 (2.3) | Self-reported and expired CO confirmed (cutoff: 10ppm).  7-day PPA 6 months: 9% (ME) vs. 10% (SC) | The abstinence rates decrease at 2, 4 and 6-months (12%-9%-9% in ME and 13%-10%-10% in SC). No between group significant differences. The nicotine patches were provided by health educators, along with brief bi-weekly counselling, which may lead to higher adherence and improving abstinence rates due to additional contacts.  Limitations: Low external validity due to only Northeastern U.S recruitment. |
|  |  | Brief counseling + NRT (n=232) | Standard care (SC): Two brief counseling sessions (5 minutes), reading materials.  Eight weeks of NRT. | Health Educator |  |  |  |
| Mercie et al. [20]  2018  France  Randomized, parallel, double-blinded, multicenter, placebo-controlled phase 3 trial | *Inclusion*: Age >= 18, confirmed HIV; daily smoking; willing to quit;  Regularly followed up at participating hospitals.  *Exclusion*:  psychoactive substance other than tobacco, depressive episode diagnosed by a psychiatrist, attempted suicide, ongoing treatment with interferon; use efavirenz for less than 3 months; had efavirenz-related adverse events; known varenicline hypersensitivity; ongoing other smoking cessation therapy, pregnant, breastfeeding. occupations requiring high vigilance; not affiliated to the health-care system. | Intensive counseling + Pharmacotherapy (n=123) | Ten to fifteen in-person counseling sessions.  Varenicline for 12 weeks. | Smoking cessation expert | Mean age, SD: 45 (9)  83% Male  Mean cigarettes per day, SD: 20 (8)  Mean FTND, SD: 5.4 (2) | Self-reported and expired CO confirmed (cutoff: 10ppm).  CA weeks 9-48: 15% (varenicline) vs 6% (placebo); OR: 2.5 (95% CI: 1.0-6.1). | Varenicline with intensive counseling showed efficacy in short-term and long-term prolonged abstinence compared to placebo with intensive counseling. Adverse events occurrence was low and reassuring.  Limitations: 34 participants dropped out after randomization may weaken ITT results. Incomplete outcome data. |
|  |  | Intensive counseling + placebo (n=125) | Ten to fifteen in-person counseling sessions.  Able to receive Varenicline after W24 if non-abstinent and motivated to quit. | Smoking cessation expert |  |  |  |
| Mussulman et al. [21]  2018  USA  Randomized controlled trial, no blinding. | *Inclusion*: Age >= 18; daily smoking; speak English or Spanish; planning to stay abstinent post discharge; no other household member enrolled; not pregnant; no health issue deemed eligible for participation. | Brief counseling + Quitline (hand-off) + NRT (n=11) | Brief counseling  Quitline warm hand-off (  Calling and transferring the call from Quitline to the patient’s bedside/ mobile phone).  NRT based on individual needs. | Hospital staff + Quitline staff | Mean age, SD: 47.7 (8.6)  48% African American  76% Male  Mean cigarettes per day, SD: 16.8 (11.4) | Self-reported and serum cotinine confirmed (cutoff: 15ng/mL).  7-day PPA 6 months: 45.50% (warm hand-off) vs. 14.30% (fax-referral). | Overall high acceptability and feasibility. Greater abstinence rates with warm hand-off compared to fax-referral (no differences observed in parent study).  Limitation: Small sample size, only including high quit motivation. |
|  |  | Brief counseling + Quitline (fax referral) + NRT(n=14) | Brief counseling  Quitline fax-referral  NRT based on individual needs. | Hospital staff + Quitline staff |  |  |  |
| O'Cleirigh et al. [22]  2018  USA  Randomized controlled trial, outcome evaluators blinded. | *Inclusion*: Age 18-65; self-reported HIV; daily smoking, willing to quit. | Intensive counseling + NRT (n=26) | QUIT program: Nine counseling sessions (60 minutes)  NRT provided at Week 6. | Trained psychology interns and postdoctoral fellows | Mean age, SD: 49.7 (7.9);  50.0 % African American, 46.2% Caucasian;  88.5% Male;  Mean cigarettes per day, SD: 14.4 (7.8)  Mean breath CO, SD: 16.2 ppm (10.8)  Mean FTND, SD: 6.5 (2.6) | Self-reported and expired CO confirmed (cutoff: 4ppm).  7-day PPA 6 months: 46% (QUIT) vs. 5% (ETAU). | Integrating anxiety/depression and smoking cessation counseling showed significantly higher 7-day PPA abstinence rates compared to standard smoking cessation counseling.  Limitations: Drop-out rates may affect sample size and statistical power, spaced counseling sessions in ETAU may reduce motivation, compensation for participants may not reflect practical settings. |
|  |  | Brief counseling + NRT (n=27) | ETAU program: One counseling session (60 minutes) and four post-quit session (10 minutes). NRT provided at Week 6. | Research staff | Mean age, SD: 51.2 (8.5);  48.1 % African American, 44.4% Caucasian;  88.5% Male;  Mean cigarettes per day, SD: 15.4 (9.7)  Mean breath CO, SD: 11.8 ppm (7.4)  Mean FTND, SD: 7.2 (2.8) |  |  |
| Parienti et al. [23]  2017  France  Non-randomized, prospective cohort study, no blinding. | *Inclusion*: confirmed HIV, interested in the program. | Intensive counseling + NRT/Pharmacotherapy (n=147) | Five counseling sessions NRT/Varenicline prescribed based on individual needs by physician. | Specialized physician | Mean age, SD: 45.5 (9.0)  66.5% Male  Mean cigarettes per day, SD: 13.4 (12.4)  Mean FTND, SD: 5.2 (2.7) | Self-reported and expired CO confirmed (cutoff: Not reported)  7-day PPA 6 months: 40.80% | Smoking prevalence among HIV-patients was high. The program was feasible; however, considerations were recommended to improve reach and retention rates of participants. |
| Shuter et al. [24]  2020  USA  Randomized control trial, open label, no blinding. | *Inclusion:* At least 365 days elapsed since enrollment, age > 18, confirmed HIV, current smoker, willing to quit, no nicotine patch contraindication | Intensive counseling + NRT (n=165) | One brief counseling session, reading materials. Eight group therapy sessions.  Twelve weeks of NRT | Trained Psychologist + PLWH Peers | Not reported. Refer to Stanton et al. (2020) for the information of the original study sample. | Self-reported and expired CO confirmed (cutoff: 10ppm).  CA 12 months: 10.3% (intensive counseling) vs. 4.2% (brief counseling). OR: 2.61 (95%CI: 1.05-6.47)  7-day PPA late follow-up (over 12 months): 12.70% (intensive counseling) vs 6.60% (brief counseling). OR: 2.06 (95%CI: 0.96-4.41). | Intensive counseling intervention group had higher long-term abstinence rates. These findings may provide evidence that smoking cessation program has long-term effects.  Limitations: High lost-to-follow rates, only include participants from 1 out of 2 study sites. |
|  |  | Brief counseling + NRT (n=166) | One brief counseling session, reading materials.  Twelve weeks of NRT |  |  |  |  |
| Shuter et al. [25]  2022  USA  Two-arm, parallel-group randomized controlled trial, outcome evaluators and investigators blinded. | *Inclusion:* Confirmed HIV; current smoker; willing to quit; internet access weekly; 6^th^ grade reading level; no contradiction; not pregnant or nursing; no other current cessation treatment; no other household member enrolled in the trial. | Intensive counseling + NRT (n=255) | Positively Smoke Free on the Web (PSFW), an interactive, multimodal web-based intervention integrated into a social network platform to support PLWH quit smoking.  Twelve weeks of NRT. | None | Mean age, SD: 50.5 (10.2)  83.5% African American  58.4% Male  Mean cigarettes per day, SD: 11.3 (8.7)  Nicotine dependence: High (11.0%), Low (17.7%), Low/Moderate (29.9%), Moderate (41.3%). | Self-reported and expired CO confirmed (cutoff: 10ppm).  7-day PPA 6 months: 14.9% (PSFW) vs. 8.8% (AHA), OR: 1.82 (95%CI: 1.04 – 3.18)  No reduction in number of smoking cigarettes among non-quitters. | Promising program consisted of HIV-tailored content and social support via online social platform. Significantly higher abstinence rates seen in PSFW vs. AHA.  Similar trends and effect sizes when analyzed for 6ppm cut-off point.  Limitations: low external validity due to study population characteristics (same geographical region, higher rate of heterosexual transmission) |
|  |  | Counseling + NRT (n=251) | An attention-matched web-based control intervention (AHA) which has one smoking-cessation module.  Twelve weeks of NRT. | None | Mean age, SD: 49.9 (10.6)  81.7% African American  56.2% Male  Mean cigarettes per day, SD: 11.8 (8.9)  Nicotine dependence: High (7.6%), Low (18.4%), Low/Moderate (24.0%), Moderate (50.0%). |  |  |
| Stanton et al. [26]  2015  USA  Randomized control trial, no blinding. | *Inclusion*: Age > 18; confirmed HIV; Latino or Hispanic; current smoker; referred by a physician.  *Exclusion*: already received smoking cessation therapy; nicotine patch contraindications; pregnant | Intensive counseling + NRT (n=154) | Aurora: Four in-person counseling sessions and 8 weeks of nicotine patches. | Health Educators | Mean age, SD: 45 (8)  56% Puerto Rican descent  64% Male | Self-reported and expired CO confirmed (cutoff: 10ppm).  7-day PPA 6 months: 8% (Aurora) vs. 11% (ESC). No between-group differences.  7-day PPA 12 months: 6% (Aurora) vs. 7% (ESC). No between-group differences.  Significant reductions in smoking intensity, number of quit attempts, nicotine dependence FTND in total sample. | No evidence that more intensive, HIV-tailored intervention improved smoking cessation efficacy compared to ESC. Low nicotine patches use adherence. Only 18% brought in a social support buddy. Suggesting alternative smoking cessation strategies.  Limitations: low generalizability study population, enhanced standard care may have attenuated Aurora's efficacy; uneven contacts in two treatments, suboptimal NRT adherence. |
|  |  | Brief counseling + NRT (n=148) | Enhanced standard care (ESC): Two in-person sessions and 8 weeks of nicotine patches. | Health Educators |  |  |  |
| Stanton et al. [27]  2020  USA  Randomized control trial, no blinding. | *Inclusion*: Age > 18; confirmed HIV; current smoking; willing to quit; willing to receive group therapy.  *Exclusion*: Pregnant; previous participation in the trial; contraindications to nicotine patch use; not meeting inclusion criteria. | Intensive counseling + NRT (n=216) | Positive Smoke Free (PSF): One brief counseling session, reading materials. Eight group therapy sessions.  Twelve weeks of NRT | Trained Psychologist + PLWH Peers | Mean age, SD: 50.3 (9.0)  65.6% African American  55.5% Male  Mean cigarettes per day, SD: 9.7 (8.1)  Mean FTND, SD: 4.6 (2.3) | Self-reported and expired CO confirmed (cutoff: 10 ppm)  7-day PPA 6 months: 13.0% (PSF) compared to 13.30% (brief counseling). OR: 0.97 (95%CI: 0.56-1.69) | PSF had a significantly higher abstinence rate at 3-month, but this effect vanished by 6-month. Comparable results with available literature, further evidence that behavioral interventions had limited long-term effect sizes.  Limitations: Low generalizability of study population, potential bias due to loss-to-follow-up, not assessed smoking behavior changes. |
|  |  | Brief counseling + NRT (n=226) | One brief counseling session, reading materials.  Twelve weeks of NRT |  | Mean age, SD: 50.8 (9.2)  66.2% African American  50.5% Male  Mean cigarettes per day, SD: 11.2 (7.7)  Mean FTND, SD: 4.9 (2.2) |  |  |
| Tindle et al. [28]  2022  Russia  4-group, randomized, double-blinded, placebo-controlled study. | Inclusion: Age 18-70; confirmed HIV; > 5 heavy drinking days; daily smoking; willing to reduce smoking/ alcohol.  Exclusion: Not speak Russian; pregnant (current/planning), nursing, received smoking cessation therapy last month; cognitive impairment; unstable psychiatric, history of seizures; allergic to study medications. | Brief counseling + Pharmacotherapy (n=100) | Twelve weeks of active varenicline  Placebo NRT | Not reported | Mean age, SD: 39 (6)  100% Caucasian  65.8% Male  Mean cigarettes per day, SD: 21 (8)  Mean breath CO, SD: 17 (9) ppm | Self-reported and expired CO confirmed (cutoff: 10ppm)  7-day PPA 6 months: 14.1% (group 1) vs. 16.3% (group 2) vs. 20.7% (group 3) vs. 20.2% (group 4).  7-day PPA 12 months: 17.7% (group 1) vs. 18.3% (group 2) vs. 25.3% (group 3) vs. 20.2% (group 4).  Reductions in number of daily smoking cigarettes: from 21 (SD:8) to 7.2-8.2 at 12 months. | The trial did not observe statistically significant differences in smoking and drinking abstinence across intervention groups. Patients in all groups reduced heavy drinking days and number of daily smoking cigarettes.  Higher adherence to pills than spray medications.  Limitations: single site, low generalizability, no placebo control. |
|  |  | Brief counseling + NRT (n=99) | Placebo varenicline  Eight weeks of mouth spray NRT contained 1 mg of nicotine per spray. | Not reported |  |  |  |
|  |  | Brief counseling + Pharmacotherapy (n=100) | Titrated cytosine for 25-day regimen  Placebo NRT | Not reported |  |  |  |
|  |  | Brief counseling + NRT (n=101) | Placebo cytosine  Eight weeks of mouth spray NRT contained 1 mg of nicotine per spray. | Not reported |  |  |  |
| ***Low-and-middle income countries*** | | | | | | | |
| Elf et al. [29]  2025  South Africa  Two-armed, open-label, randomized controlled trial | *Inclusion:* Age >= 18; confirmed HIV; current cigarette smoker; willing to quit.  *Exclusion:* Pregnancy or breastfeeding; unstable medical conditions; contraindications to nicotine patch therapy; concurrent use of other smoking cessation pharmacotherapy, e-cigarettes, or smokeless tobacco | Behavioral counseling (n=281) | Brief counseling at baseline assessment + Self-help materials | Study staff | Median age, IQR: 37 (31, 46)  78% Male  Median cigarettes per day, IQR: 10 (5-15)  Median expired CO, IQR: 15 ppm (9-22) | Self-reported, Expired CO (cutoff: 7 pp) and urine cotinine (cutoff: 0.4 µg/mL) confirmed.  7-day PPA at 6 months:  BC + c-NRT: 15%  BC: 10% | Among participants who continued to smoke, the c-NRT arm showed larger reductions in exhaled breath CO and increased quit motivation, c-NRT did not have statistically better cessation outcomes compared to intensive counseling at 6 months. Differences in verified abstinence (15% vs 10%) were not statistically significant, though effect sizes matched population norms. High patch adherence suggested the intervention is feasible.  Limitations: open-label design introduced potential bias; single peri-urban setting limited generalizability; excluded participants not ready to quit |
|  |  | Behavioral counseling + c-NRT (n=280) | Brief counseling + Self-help materials + 12 weeks nicotine gum or nicotine patch |  |  |  |  |
| Himelhoch et al. [30]  2024  Kenya  Factorial design (2x2), randomized, SoC arm participants and outcome evaluators blinded, | *Inclusion:* HIV diagnosis; self-reported smoking ≥ 1 cigarette/day; exhaled CO ≥5 ppm; motivated to quit within next 6 months (Ladder score 6-8); able to speak/write English or Swahili.  *Exclusion:* Bupropion use in past 3 months; allergic reaction/ hypersensitivity to bupropion; current/planned pregnancy or nursing; medically unstable; moderate/severe substance use disorder. | PSF + bupropion (n=74) | 8 intensive counseling sessions + 12 weeks bupropion | PSF: Trained providers supervised by a trial psychologist.  SOC: Trained trial staff. | Mean age, SD: 42.5 (10.2)  71.4% Male  99.7% African  Mean cigarettes per day, SD: 10.7 (7.2)  Mean FTND’s score, SD: 4.5 (2.3) | Exhaled CO (cutoff: 7 ppm) confirmed.  7-day PPA at 36 weeks:  PSF + bupropion: 38.9%  PSF + placebo: 20.3%  SoC + bupropion: 23.6%  SoC + placebo: 6.6% | Both Bupropion and PSF demonstrated significantly higher abstinence rates at 36 weeks compared to controls. Combined therapy (38.9%) showed additive effects over single modalities. Female sex was associated with higher quit rates.  Bupropion group reported significantly higher rates of excessive sweating.  Limitations: Single urban site limited generalizability; SOC control did not account for attention bias; lack of viral load data; lack of biological confirmation for medication adherence; bupropion market availability issues. |
|  |  | PSF + placebo (n=76) | 8 intensive counseling sessions + 12 weeks placebo |  |  |  |  |
|  |  | SOC + bupropion (n=74) | Brief advice + 12 weeks bupropion |  |  |  |  |
|  |  | SOC + placebo (n=76) | Brief advice + 12 weeks placebo |  |  |  |  |
| Keke et al. [31]  2026  South Africa  Secondary RCT (retreatment phase), open-label. | *Inclusion:* Daily cigarette smoking PLWH, failing to achieve abstinence at trial follow-up at 6 months. | Repeat Behavioral counseling (n=196) | Re-treatment after failing the first round of treatment.  Behavioral counseling (BC): 4 intensive counseling sessions.  BC + c-NRT: 4 sessions + 10-week nicotine patches and gum. | Study staff | Median age, IQR: 37 (31-46)  78% Male  Median breath CO, IQR: 16 (10-22)  Moderate/High heaviness smoking index: 77% | Self-reported, Expired CO (cutoff: 7 pp) and urine cotinine (cutoff: 0.4 µg/mL) confirmed.  Biochemically confirmed abstinence at 6 months after re-treatment:  BC: 8%  BC + c-NRT: 11% | Findings: Adding cNRT to counseling did not significantly improve retreatment, but the repeated care model yielded additional quitters.  Limitations: Exclusion of loss to follow-up participants after first round. Self-reported and low medication adherence. No comparison group who did not receive retreatment. Secondary analysis which was not planned and underpowered to detect statistically significant differences. |
|  |  | Repeat Behavioral counseling + c-NRT (n=188) |  |  |  |  |  |
| Shelley et al. [32]  2026  Vietnam  Open-label, three-arm pragmatic, randomized controlled trial | *Inclusion:* Age >= 18; current cigarette smoker; lived in Hanoi; visit HIV outpatient clinic in the last 12 months; daily access to mobile phone.  *Exclusion:* Concurrent use of other smoking cessation program; contradiction to nicotine gum; Pregnancy or breastfeeding. | Quitline referral (n=221) | Brief counseling + 10 counseling sessions through telephone | Trained physician for brief counseling.  Trained nurses for intensive counseling.  Quitline counselors for telephone counseling by National Quitline services | Mean age, SD: 44.4 (7.1)  96% Male  Mean cigarettes per day, SD: 14.5 (8.2)  Mean FTND score, SD: 4.9 (2.4) | Self-reported, Expired CO 7-day PPA (cutoff: 8 ppm) confirmed.  7-day PPA at 6 months (biochemically confirmed):  Quitline referral: 13%  Counselling+SMS: 18%  Counselling+SMS  +gum: 18% | No significant differences in 6-month biochemically confirmed smoking abstinence were observed across the three intervention arms. Combining nicotine gum with tailored behavioral counseling did not yield higher abstinence rates than tailored counseling alone. However, integrating routine screening, tailored nurse-delivered counseling, and proactive Quitline referrals were highly feasible within HIV outpatient care. Utilizing existing national Quitline infrastructure serves as a practical, resource-efficient strategy for HIV care systems in low-and-middle-income countries. Dual users of cigarettes and waterpipes exhibited lower overall quit rates than exclusive cigarette smokers.  Limitations: The trial may have been underpowered to detect small but clinically relevant group differences; the Quitline arm offered a greater number of sessions (up to 10) over a potentially longer timeframe (up to 12 months) compared to the tailored arms (6 sessions over 4.5 months); unable to objectively verify consistent adherence to the nicotine gum; resource constraints precluded the use of combination NRT, which is generally more effective than a single NRT product; high refusal rates among eligible patients suggests the enrolled cohort may represent individuals with higher baseline quit motivation. |
|  |  | Counselling+SMS (n=225) | Brief counseling + 6 intensive counseling sessions + 12 weeks daily SMS support |  |  |  |  |
|  |  | Counselling+SMS+gum (n=226) | Brief counseling + 6 intensive counseling sessions + 12 weeks daily SMS support + 6 weeks of nicotine gum |  |  |  |  |

*** *Abbreviations: ALT, alanine aminotransferase; ART, antiretroviral therapy; AST, aspartate aminotransferase; BMI, body mass index; CA, continuous abstinence; CD4, cluster of differentiation 4 (T-cell count); CI, confidence interval; CO, carbon monoxide (eCO, exhaled CO); FTND, Fagerström Test for Nicotine Dependence; HIV, human immunodeficiency virus; IQR, interquartile range; NRT, nicotine replacement therapy; OR, odds ratio; PLWH, people living with HIV; PPA, point-prevalence abstinence; ppm, parts per million; PSF, Positively Smoke Free; SC, standard care; SD, standard deviation.*

**References**

[1] Altobelli D, Ricci E, Maggi P, et al. Smoking Cessation in People Living With HIV: Results From Italian STOPSHIV Project Cohort. 2026; 101: 441–448.

[2] Ashare RL, Thompson M, Serrano K, et al. Placebo-controlled randomized clinical trial testing the efficacy and safety of varenicline for smokers with HIV. *Drug Alcohol Depend* 2019; 200: 26‐33.

[3] Balfour L, Wiebe SA, Cameron WD, et al. An HIV-tailored quit-smoking counselling pilot intervention targeting depressive symptoms plus Nicotine Replacement Therapy. *AIDS Care* 2017; 29: 24–31.

[4] Bui TC, Piñeiro B, Vidrine DJ, et al. Quitline Treatment Enrollment and Cessation Outcomes Among Smokers Linked With Treatment via Ask-Advise-Connect: Comparisons Among Smokers With and Without HIV. *Nicotine Tob Res* 2020; 22: 1640–1643.

[5] Chew D, Steinberg MB, Thomas P, et al. Evaluation of a Smoking Cessation Program for HIV Infected Individuals in an Urban HIV Clinic: Challenges and Lessons Learned. *AIDS Res Treat* 2014; 2014: 237834.

[6] Cioe PA, Pinkston M, Stang GS, et al. Peer Navigation for Smoking Cessation in People With HIV Who Smoke: A Pilot Randomized Controlled Trial. *Nicotine Tob Res* 2025; 27: 517–524.

[7] Cui Q, Robinson L, Elston D, et al. Safety and tolerability of varenicline tartrate (Champix(®)/Chantix(®)) for smoking cessation in HIV-infected subjects: a pilot open-label study. *AIDS Patient Care STDS* 2012; 26: 12–19.

[8] De Socio GV, Pasqualini M, Ricci E, et al. Smoking habits in HIV-infected people compared with the general population in Italy: a cross-sectional study. *BMC Public Health* 2020; 20: 734.

[9] Edelman EJ, Deng Y, Dziura J, et al. Clinical Pharmacists, Medications, and Contingency Management for Targeting Smoking in HIV Clinics: A Randomized Clinical Trial. *JAMA Netw Open* 2026; 9: e2560593.

[10] Edwards S, Puljević C, Dean JA, et al. Tobacco Harm Reduction with Vaporised Nicotine (THRiVe): A Feasibility Trial of Nicotine Vaping Products for Smoking Cessation Among People Living with HIV. *AIDS Behav*. Epub ahead of print 22 July 2022. DOI: 10.1007/s10461-022-03797-0.

[11] Elzi L, Spoerl D, Voggensperger J, et al. A smoking cessation programme in HIV-infected individuals: a pilot study. *Antivir Ther* 2006; 11: 787–795.

[12] Fitzgerald SA, Richter KP, Mussulman L, et al. Improving Quality of Care for Hospitalized Smokers with HIV: Tobacco Dependence Treatment Referral and Utilization. *Jt Comm J Qual Patient Saf* 2016; 42: 219–224.

[13] Fuster M, Estrada V, Fernandez-Pinilla MC, et al. Smoking cessation in HIV patients: rate of success and associated factors. *HIV Med* 2009; 10: 614–619.

[14] Grabovac I, Brath H, Schalk H, et al. Clinical setting-based smoking cessation programme and the quality of life in people living with HIV in Austria and Germany. *Qual Life Res* 2017; 26: 2387–2395.

[15] Gritz ER, Danysh HE, Fletcher FE, et al. Long-term outcomes of a cell phone-delivered intervention for smokers living with HIV/AIDS. *Clin Infect Dis* 2013; 57: 608‐615.

[16] Himelhoch S, Kelly D, deFilippi C, et al. Optimizing behavioral and pharmacological smoking cessation interventions among people with HIV. *AIDS* 2024; 38: 669–678.

[17] Humfleet GL, Hall SM, Delucchi KL, et al. A randomized clinical trial of smoking cessation treatments provided in HIV clinical care settings. *Nicotine Tob Res* 2013; 15: 1436‐1445.

[18] Kim SS, Darwish S, Lee SA, et al. A randomized controlled pilot trial of a smoking cessation intervention for US women living with HIV: telephone-based video call vs voice call. *Int J Womens Health* 2018; 10: 545–555.

[19] Lloyd-Richardson EE, Stanton CA, Papandonatos GD, et al. Motivation and patch treatment for HIV+ smokers: a randomized controlled trial. *Addict Abingdon Engl* 2009; 104: 1891‐1900.

[20] Mercié P, Arsandaux J, Katlama C, et al. Efficacy and safety of varenicline for smoking cessation in people living with HIV in France (ANRS 144 Inter-ACTIV): a randomised controlled phase 3 clinical trial. *Lancet HIV* 2018; 5: e126–e135.

[21] Mussulman LM, Faseru B, Fitzgerald S, et al. A randomized, controlled pilot study of warm handoff versus fax referral for hospital-initiated smoking cessation among people living with HIV/AIDS. *Addict Behav* 2018; 78: 205‐208.

[22] O’Cleirigh C, Zvolensky MJ, Smits JAJ, et al. Integrated treatment for smoking cessation, anxiety, and depressed mood in people living with HIV: A randomized controlled trial. *J Acquir Immune Defic Syndr* 2018; 79: 261–268.

[23] Parienti JJ, Merzougui Z, De La Blanchardière A, et al. A Pilot Study of Tobacco Screening and Referral for Smoking Cessation Program among HIV-Infected Patients in France. *J Int Assoc Provid AIDS Care* 2017; 16: 467–474.

[24] Shuter J, Kim RS, Durant S, et al. Brief Report: Long-Term Follow-up of Smokers Living With HIV After an Intensive Behavioral Tobacco Treatment Intervention. *J Acquir Immune Defic Syndr* 2020; 84: 208–212.

[25] Shuter J, Chander G, Graham AL, et al. A randomized trial of a web-based tobacco treatment and online community support for people with HIV attempting to quit smoking cigarettes. *J Acquir Immune Defic Syndr* 2022; 90: 223–231.

[26] Stanton CA, Papandonatos GD, Shuter J, et al. Outcomes of a Tailored Intervention for Cigarette Smoking Cessation Among Latinos Living With HIV/AIDS. *Nicotine Tob Res* 2015; 17: 975‐982.

[27] Stanton CA, Kumar PN, Moadel AB, et al. A Multicenter Randomized Controlled Trial of Intensive Group Therapy for Tobacco Treatment in HIV-Infected Cigarette Smokers. *J Acquir Immune Defic Syndr 1999* 2020; 83: 405‐414.

[28] Tindle AH, Freiberg M, Cheng D, et al. Effectiveness of Varenicline and Cytisine for Alcohol Use Reduction Among People With HIV and Substance Use: A Randomized Clinical Trial. *JAMA Netw Open* 2022; 5: e2225129.

[29] Elf JL, Lebina L, Motlhaoleng K, et al. A randomized trial for combination nicotine replacement therapy for smoking cessation among people with HIV in a low-resourced setting. *AIDS* 2025; 39: 526–534.

[30] Himelhoch SS, Koech E, Omanya AA, et al. Efficacy of Smoking Cessation Interventions among People with HIV in Kenya. *NEJM Evid*; 3. Epub ahead of print 22 October 2024. DOI: 10.1056/EVIDoa2400090.

[31] Keke C, Lebina L, Motlhaoleng K, et al. Repeat Behavioral Counseling, With and Without Combination Nicotine Replacement Therapy, for Smoking Cessation Among People With HIV in South Africa. *AIDS Behav*. Epub ahead of print 31 January 2026. DOI: 10.1007/s10461-026-05064-y.

[32] Shelley D, Armstrong-Hough M, Nguyen T, et al. Effectiveness of behavioural tobacco cessation interventions with and without pharmacotherapy among people living with HIV in Viet Nam: a three-arm pragmatic randomised controlled trial. *Lancet Glob Health* 2026; 14: e407–e416.
