# Supplementary material for: Integrating smoking cessation into HIV care settings: A systematic review and meta-analysis of effectiveness and the evidence gap in cost-effectiveness
Source: PLoS One. 2026 Jul 30;21(7):e0350040. doi: 10.1371/journal.pone.0350040 (PMC13423040; doi:10.1371/journal.pone.0350040)
Supplement: S3 Table — (DOCX) [file pone.0350040.s007.docx]

**S3 Table. Excluded studies from low- and middle-income countries**

| **Author**  **Year**  **Country**  **Study design** | **Eligible criteria** | **Service group, sample size** | **Description** | **Counseling**  **person** | **Participants characteristics** | **Outcome measures and abstinence rates** | **Findings and limitations** |
| --- | --- | --- | --- | --- | --- | --- | --- |
| ***Excluded studies from low- and middle-income countries*** | | | | | | | |
| Bui et al. [1]  2022  Cambodia  Mixed-methods, Two-group, single-blind, randomized controlled trial. | *Inclusion:* Age ≥ 18 years, HIV-positive, Smoke ≥ 5 cigarettes/day and ≥ 100 cigarettes lifetime; Set quit date within two weeks, Able to read Khmer.  *Exclusion:* Ineligible on medical or psychiatric condition deemed by physician/clinician, Enrolled in another cessation study. | Standard care (SC)  (n=25) | One brief advice at baseline and self-help reading material (adapted for Khmer). Access to mHealth smartphone application. | Research staff | SC: Mean age, SD: 44.9 (6.9); 100% Male; Number of cigarettes/day, mean (SD): 13.5 (7.9)  AM: Mean age, SD: 43.4 (7.6); 100% Male; Number of cigarettes/day, mean (SD): 12.7 (6.9) | 7-day PPA, self-reported and expired CO confirmed (<5 ppm)  2 months:  SC: 8%  AM: 40% | The approach using mHealth was highly acceptable and feasible. The AM arm demonstrated efficacy compated to SC.  Limitations: Short follow-up period, small sample size. Lack of information on tobacco use and drug use history. |
|  |  | Automated Messaging (AM)  (n=25) | Standard care components plus proactive personalized messages for eight weeks. |  |  |  |  |
| Kumar et al. [2]  2017  India  Randomized controlled trial. | *Inclusion*: Male patients, age >18 years, diagnosed with either TB or HIV (HIV group included HIV/TB co-infection), and a history of current smoking (at least one cigarette in the past 1 week) | Intervention group: Physician’s advice + standard counseling + brochures (n=80) | Modified 5As strategy (Ask, Advise, Assess, Assist, Arrange); brief structured advice to patient and family; quit date setting; education on HIV/TB-specific smoking harms | Physicians and trained counselors (medical social workers) | Mean age, SD: 39.4 (8.5)  100% male  35% high nicotine dependence  Mean cigarettes/bidis per da, SD: 14.3 (10.8) | 1-month self-reported abstinence confirmed by expired CO <10 ppm  Intervention: 41%  Comparator: 35% | Physician-led advice is feasible and acceptable for TB and HIV patients.  Limitations: Short 1-month follow-up; small sample size; restricted to male participants. |
|  |  | Comparator group: Standard counseling + brochures (n=80) | Pictorial brochures on smoking complications; 15–20 minute standard counseling session based on the National Tobacco Control Programme of India |  |  |  |  |
| Poudel et al. [3]  2023  Nepal  Single-arm pilot study. | *Inclusion:* HIV positive, ≥18 years, smoked ≥100 cigarettes lifetime and currently ≥5/day, willing to set quit date within one month, own a video-capable smartphone | Video-based smoking cessation intervention (n=48) | Eleven 3–8 minute video sessions in Nepali based on the Phase-Based Model (PBM); focused on preparation (motivation), cessation (managing triggers/cues), and maintenance (relapse prevention); delivered via email links | Research assistants and video-delivered content | Mean age, SD: 36.6 (9.9)  73% Male  Mean cigarettes/day, SD: 18.3 (9.2)  71% High/Very high nicotine dependence | 1-week point prevalence abstinence (PPA) at 3 months, self-report confirmed by CO <5 ppm  Abstinence rate: 39.6% | High feasibility and acceptability (96% watched all videos; 100% retention).  Limitations: Single-arm (no comparison group); small sample size; short follow-up period. |
| Tsima et al. [4]  2020  Botswana  Single-arm pilot trial. | *Inclusion:* HIV positive, Age 18-65 years, smoke ≥ 5 cigarettes/day.  *Exclusion:* Untreated alcohol dependence, current use of cessation meds, unstable depression/psychosis, use of smokeless tobacco, or participation in other programs | Behavioural Activation/Problem Solving for Smoking Cessation (BAPS-SC) (n=40) | Five-session counseling model merging behavioural activation (increasing healthy rewarding activities) and problem-solving therapy; sessions conducted via telephone over 12 weeks | Trained research assistants/recruiters | Median age, IQR: 39.5 [34,48]  95% Male  Median cigarettes/day, IQR: 10 [6,11.5]  92.5% Moderate nicotine dependence  7.5% High nicotine dependence | 7-day PPA at 12 weeks, self-report confirmed by CO <8 ppm  Abstinence rate: 37.5% | Feasible and appealing to HIV-infected smokers; leverages existing HIV infrastructure.  Limitations: Single-arm design; low retention (53%); follow-up limited to 12 weeks |

** Abbreviations: CO, carbon monoxide; HIV, human immunodeficiency virus; IQR, interquartile range; PPA, point-prevalence abstinence; ppm, parts per million; SC, standard care; SD, standard deviation*

**References**

[1] Bui TC, Sopheab H, Businelle MS, et al. Mobile-health intervention for smoking cessation among Cambodian people living with HIV: A mixed-methods pilot study. *AIDS Care* 2022; 34: 430–439.

[2] Kumar SR, Pooranagangadevi N, Rajendran M, et al. Physician’s advice on quitting smoking in HIV and TB patients in south India: a randomised clinical trial. *Public Health Action* 2017; 7: 39–45.

[3] Poudel KC, Poudel-Tandukar K, Silwal RC, et al. Feasibility, Acceptability, and Preliminary Effects of a Video-Based Intervention for Smoking Cessation Among People with HIV in Kathmandu, Nepal: A Single-Armed Pilot Study. *AIDS Behav* 2023; 27: 3468–3477.

[4] Tsima BM, Moedi P, Maunge J, et al. Feasibility of implementing a novel behavioural smoking cessation intervention amongst human immunodeficiency virus-infected smokers in a resource-limited setting: A single-arm pilot trial. *South Afr J HIV Med*; 21. Epub ahead of print 24 June 2020. DOI: 10.4102/sajhivmed.v21i1.1075.
