# Supplementary material for: Integrating smoking cessation into HIV care settings: A systematic review and meta-analysis of effectiveness and the evidence gap in cost-effectiveness
Source: PLoS One. 2026 Jul 30;21(7):e0350040. doi: 10.1371/journal.pone.0350040 (PMC13423040; doi:10.1371/journal.pone.0350040)
Supplement: S4 Table — (DOCX) [file pone.0350040.s008.docx]

**S4 Table. Summary of findings for non-randomized studies and additional subgroup comparisons**

| **Outcomes/ Comparisons** | **Assumed risk (Placebo/ Standard care)** | **Risk difference with intervention*** | **Relative effect (95% CI)** | **No. of Participants (studies)** | **Certainty of Evidence (GRADE)** |
| --- | --- | --- | --- | --- | --- |
| ***Observational studies*** | | | | | |
| Intensive counseling + NRT vs. No intervention *(Continuous abstinence >12 months)* | 70 per 1,000 | 249 more per 1,000  (104 more to 449 more) | **RR 4.55****  (2.49 to 7.41) | 417  (1 study)[1] | ⊕⊖⊖⊖  **Very low^a,b,c^** |
| Intensive/Standard counseling vs. Brief/Soft counseling *(Abstinence at ≥6 months)* | Varies  (41 to 147 per 1,000) | Not pooled | Not pooled | 675  (2 studies)[2, 3] | ⊕⊖⊖⊖  **Very low^a,d,f^** |
| Smoking cessation interventions (Single-Arm Cohorts)  *(Abstinence at ≥6 months)* | N/A  (No control group) | N/A | Not estimable | 682  (8 studies)[4–11] | ⊕⊖⊖⊖  **Very low^a,e^** |
| ***Subgroup and other comparisons*** | | | | | |
| Bupropion vs. Placebo  *(7-day PPA)* | 132 per 1,000 | 172 more per 1,000  (54 more to 363 more) | **RR 2.36**  (1.47 to 3.79) | 300  (1 study) [12] | ⊕⊕⊕⊖  **Moderate^h^** |
| Varenicline vs. Placebo  *(Abstinence at ≥6 months)* | 65 per 1,000 | 57 more per 1,000  (8 more to 138 more) | **RR 1.92**  (1.16 to 3.17) | 610  (3 studies) [13–15] | ⊕⊕⊕⊖  **Moderate^i^** |
| NRT vs. Counseling  *(7-day PPA)* | 100 per 1,000 | 47 more per 1,000  (6 fewer to 131 more) | **RR 1.47**  (0.94 to 2.31) | 561  (1 study) [16] | ⊕⊕⊖⊖  **Low^g,j^** |
| Pharmacotherapy with/without contingency management  *(7-day PPA)* | 98 per 1,000 | 126 more per 1,000  (32 more to 290 more) | **RR: 2.29****  (1.33 to 3.96) | 323  (1 study) [17] | ⊕⊕⊖⊖  **Low^h,i^** |
| Repeat combination NRT vs. Repeat counseling alone (for prior treatment failures)  *(7-day PPA)* | 77 per 1,000 | 12 more per 1,000 (47 fewer to 190 more) | **RR: 1.16,**  (0.39 to 3.47) | 384  (1 study) [18] | ⊕⊕⊖⊖  **Low^g,j^** |

*Abbreviations: CI, confidence interval; EPHPP, Effective Public Health Practice Project; GRADE, Grading of Recommendations Assessment, Development and Evaluation; NRT, nicotine replacement therapy; OIS, optimal information size; OR, odds ratio; PPA, point-prevalence abstinence; RR, risk ratio.*

**GRADE Working Group grades of evidence:**

**High quality:** We are very confident that the true effect lies close to that of the estimate of the effect.

**Moderate quality:** We are moderately confident in the effect estimate: The true effect is likely to be close to the estimate of the effect, but there is a possibility that it is substantially different.

**Low quality:** Our confidence in the effect estimate is limited: The true effect may be substantially different from the estimate of the effect.

**Very low quality:** We have very little confidence in the effect estimate: The true effect is likely to be substantially different from the estimate of effect.

**Notes.**

*The risk in the intervention group (and its 95% confidence interval) is based on the assumed risk in the comparison group and the relative effect of the intervention (and its 95% CI).

**Elzi et al. (2006) reported an adjusted Odds Ratio (OR 6.20, 95% CI 2.80–14.30). In accordance with GRADE guidelines, this was mathematically converted to a Risk Ratio (RR) based on the control group's baseline event rate of 7% to provide a standardized relative effect measure.

**^a^**Starting with Low certainty, as the evidence is derived from observational and non-randomized studies.

**^b^**Downgraded one level for study limitation: Study exhibited a high (Weak) risk of bias according to the EPHPP tool, particularly concerning unblinded assessment and failure to control for confounders.

**^c^**Downgraded one level for imprecision: Evidence generated from a single study with a relatively low total number of quit events.

**^d^**Downgraded for inconsistency and imprecision: Studies exhibited variable baseline risks and differing point estimates, precluding appropriate statistical pooling.

**^e^**Downgraded to Very Low certainty due to very serious study limitations (complete absence of a comparator control arm across studies), extreme inconsistency in reported abstinence rates (ranging from 4.2% to 42.0%), and severe imprecision resulting from small, single-group sample sizes.

**^f^**Downgraded one level for risk of bias (study limitations): The definitive cessation outcome in the Altobelli et al. (2025) study was entirely self-reported without biochemical verification, introducing a high risk of reporting bias.

**^g^**Downgraded one level for study limitations: Open-label/unblinded study design poses a risk of performance bias.

**^h^**Downgraded one level for imprecision: Evidence generated from a single randomized controlled trial.

**^i^**Downgraded one level for imprecision: The total number of events does not meet the optimal information size (OIS) criteria.

**^j^**Downgraded one level for imprecision: The 95% confidence interval overlaps no effect (crosses 1.0) and fails to exclude important benefit or harm.

**References**

[1] Elzi L, Spoerl D, Voggensperger J, et al. A smoking cessation programme in HIV-infected individuals: a pilot study. *Antivir Ther* 2006; 11: 787–795.

[2] Altobelli D, Ricci E, Maggi P, et al. Smoking Cessation in People Living With HIV: Results From Italian STOPSHIV Project Cohort. 2026; 101: 441–448.

[3] Grabovac I, Brath H, Schalk H, et al. Clinical setting-based smoking cessation programme and the quality of life in people living with HIV in Austria and Germany. *Qual Life Res* 2017; 26: 2387–2395.

[4] Balfour L, Wiebe SA, Cameron WD, et al. An HIV-tailored quit-smoking counselling pilot intervention targeting depressive symptoms plus Nicotine Replacement Therapy. *AIDS Care* 2017; 29: 24–31.

[5] Bui TC, Piñeiro B, Vidrine DJ, et al. Quitline Treatment Enrollment and Cessation Outcomes Among Smokers Linked With Treatment via Ask-Advise-Connect: Comparisons Among Smokers With and Without HIV. *Nicotine Tob Res* 2020; 22: 1640–1643.

[6] Chew D, Steinberg MB, Thomas P, et al. Evaluation of a Smoking Cessation Program for HIV Infected Individuals in an Urban HIV Clinic: Challenges and Lessons Learned. *AIDS Res Treat* 2014; 2014: 237834.

[7] Cui Q, Robinson L, Elston D, et al. Safety and tolerability of varenicline tartrate (Champix(®)/Chantix(®)) for smoking cessation in HIV-infected subjects: a pilot open-label study. *AIDS Patient Care STDS* 2012; 26: 12–19.

[8] Edwards S, Puljević C, Dean JA, et al. Tobacco Harm Reduction with Vaporised Nicotine (THRiVe): A Feasibility Trial of Nicotine Vaping Products for Smoking Cessation Among People Living with HIV. *AIDS Behav*. Epub ahead of print 22 July 2022. DOI: 10.1007/s10461-022-03797-0.

[9] Fitzgerald SA, Richter KP, Mussulman L, et al. Improving Quality of Care for Hospitalized Smokers with HIV: Tobacco Dependence Treatment Referral and Utilization. *Jt Comm J Qual Patient Saf* 2016; 42: 219–224.

[10] Fuster M, Estrada V, Fernandez-Pinilla MC, et al. Smoking cessation in HIV patients: rate of success and associated factors. *HIV Med* 2009; 10: 614–619.

[11] Parienti JJ, Merzougui Z, De La Blanchardière A, et al. A Pilot Study of Tobacco Screening and Referral for Smoking Cessation Program among HIV-Infected Patients in France. *J Int Assoc Provid AIDS Care* 2017; 16: 467–474.

[12] Himelhoch SS, Koech E, Omanya AA, et al. Efficacy of Smoking Cessation Interventions among People with HIV in Kenya. *NEJM Evid*; 3. Epub ahead of print 22 October 2024. DOI: 10.1056/EVIDoa2400090.

[13] Ashare RL, Thompson M, Serrano K, et al. Placebo-controlled randomized clinical trial testing the efficacy and safety of varenicline for smokers with HIV. *Drug Alcohol Depend* 2019; 200: 26‐33.

[14] Himelhoch S, Kelly D, deFilippi C, et al. Optimizing behavioral and pharmacological smoking cessation interventions among people with HIV. *AIDS* 2024; 38: 669–678.

[15] Mercié P, Arsandaux J, Katlama C, et al. Efficacy and safety of varenicline for smoking cessation in people living with HIV in France (ANRS 144 Inter-ACTIV): a randomised controlled phase 3 clinical trial. *Lancet HIV* 2018; 5: e126–e135.

[16] Elf JL, Lebina L, Motlhaoleng K, et al. A randomized trial for combination nicotine replacement therapy for smoking cessation among people with HIV in a low-resourced setting. *AIDS* 2025; 39: 526–534.

[17] Edelman EJ, Deng Y, Dziura J, et al. Clinical Pharmacists, Medications, and Contingency Management for Targeting Smoking in HIV Clinics: A Randomized Clinical Trial. *JAMA Netw Open* 2026; 9: e2560593.

[18] Keke C, Lebina L, Motlhaoleng K, et al. Repeat Behavioral Counseling, With and Without Combination Nicotine Replacement Therapy, for Smoking Cessation Among People With HIV in South Africa. *AIDS Behav*. Epub ahead of print 31 January 2026. DOI: 10.1007/s10461-026-05064-y.
