# Supplementary material for: Integrating smoking cessation into HIV care settings: A systematic review and meta-analysis of effectiveness and the evidence gap in cost-effectiveness
Source: PLoS One. 2026 Jul 30;21(7):e0350040. doi: 10.1371/journal.pone.0350040 (PMC13423040; doi:10.1371/journal.pone.0350040)
Supplement: S5 Table — (DOCX) [file pone.0350040.s009.docx]

**S5 Table. Sensitivity analysis results for comparison groups.**

| **Comparison** | **Subgroup** | **Studies (k)** | **Paule-Mandel Random-Effects model** | **Fixed-Effect model** | **Modified HKSJ model** |
| --- | --- | --- | --- | --- | --- |
| Pharmacotherapy vs. Placebo/SoC | Overall | 5 | 1.86  [1.42, 2.45] | 1.86  [1.42, 2.45] | 1.86  [1.26, 2.75] |
|  | *Varenicline vs. Placebo* | 3 | 1.92  [1.16, 3.17] | 1.92  [1.16, 3.17] | 1.92  [0.64, 5.78] |
|  | *Bupropion vs. Placebo* | 1 | 2.36  [1.47, 3.79] | 2.36  [1.47, 3.79] | 2.36  [1.47, 3.79] |
|  | *NRT vs. SoC* | 1 | 1.47  [0.94, 2.31] | 1.47  [0.94, 2.31] | 1.47  [0.94, 2.31] |
| Tailored/intensive counselling vs. Brief/standard counselling | Overall | 10 | 1.34  [1.05, 1.71] | 1.35  [1.13, 1.61] | 1.34  [1.01, 1.78] |
|  | *Only PPA outcomes* | 9 | 1.29  [1.02, 1.64] | 1.32  [1.10, 1.58] | 1.29  [0.97, 1.71] |
|  | *Only CA outcomes* | 1 | 2.44  [1.04, 5.74] | 2.44  [1.04, 5.74] | 2.44  [1.04, 5.74] |
| Peer navigation/social support vs. SoC | Overall | 2 | 1.67  [0.95, 2.94] | 1.67  [0.95, 2.94] | 1.67  [0.04, 66.15] |
| System process and mode of delivery | Overall | 2 | 4.16  [1.29, 13.37] | 4.16  [1.29, 13.37] | 4.16  [0.00, 8093.03] |

** Abbreviations: CA, continuous abstinence; HKSJ, Hartung–Knapp–Sidik–Jonkman; NRT, nicotine replacement therapy; PPA, point-prevalence abstinence.*
