# Supplementary figures and images for: Integrating smoking cessation into HIV care settings: A systematic review and meta-analysis of effectiveness and the evidence gap in cost-effectiveness
Source: PLoS One. 2026 Jul 30;21(7):e0350040. doi: 10.1371/journal.pone.0350040 (PMC13423040; doi:10.1371/journal.pone.0350040)

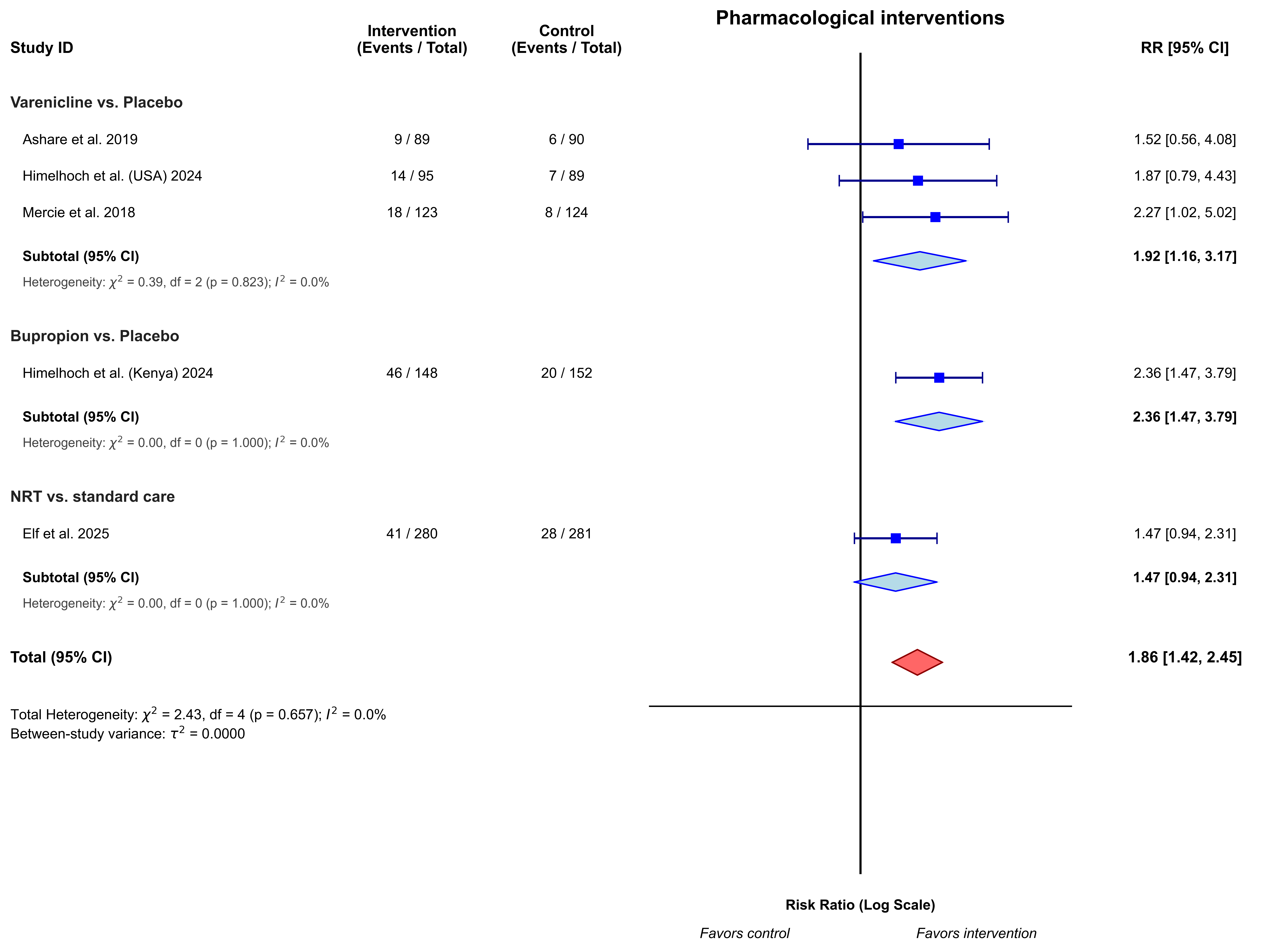

Supplement: S1 Fig — (TIF) [file pone.0350040.s010.tif]

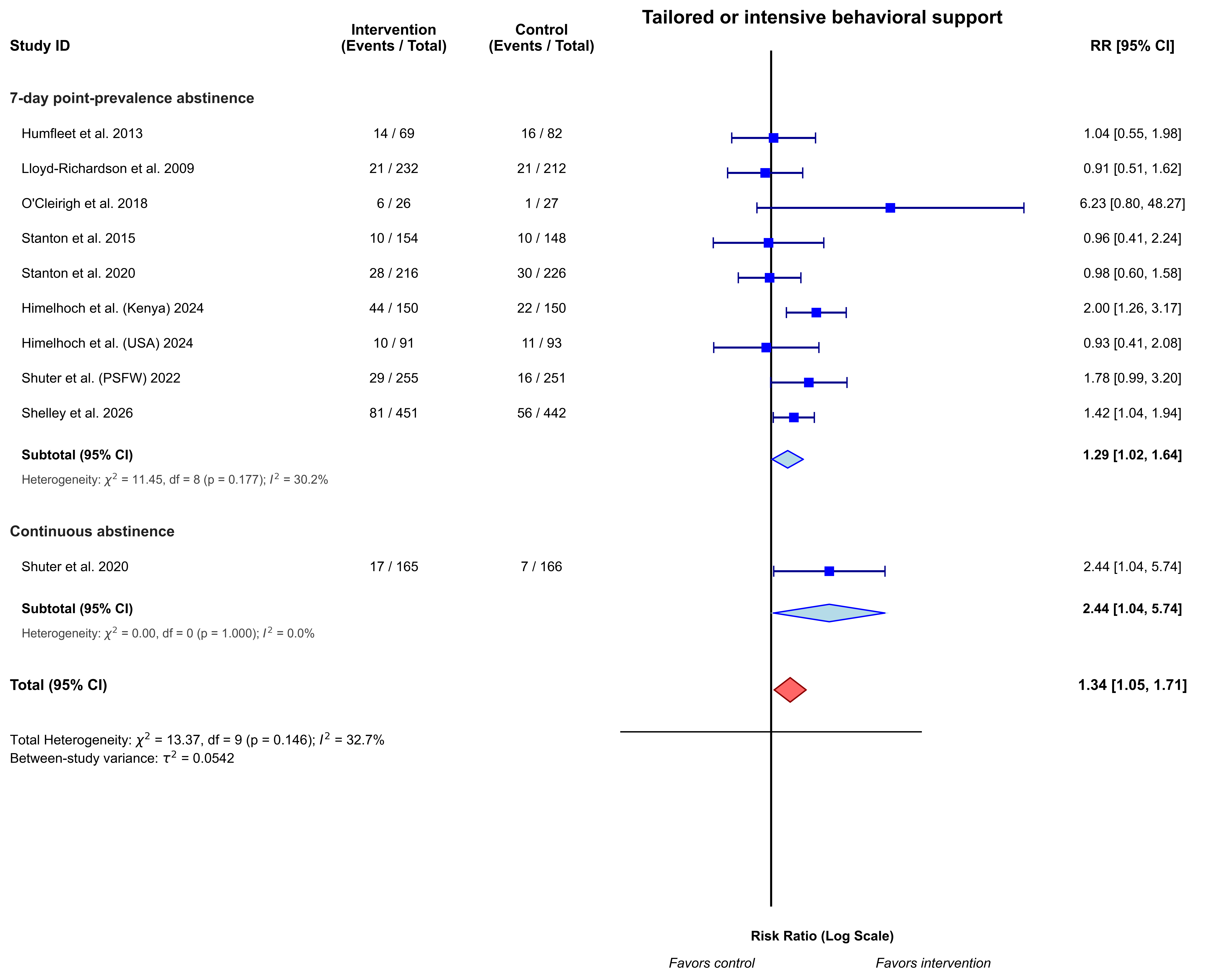

Supplement: S2 Fig — (TIF) [file pone.0350040.s011.tif]

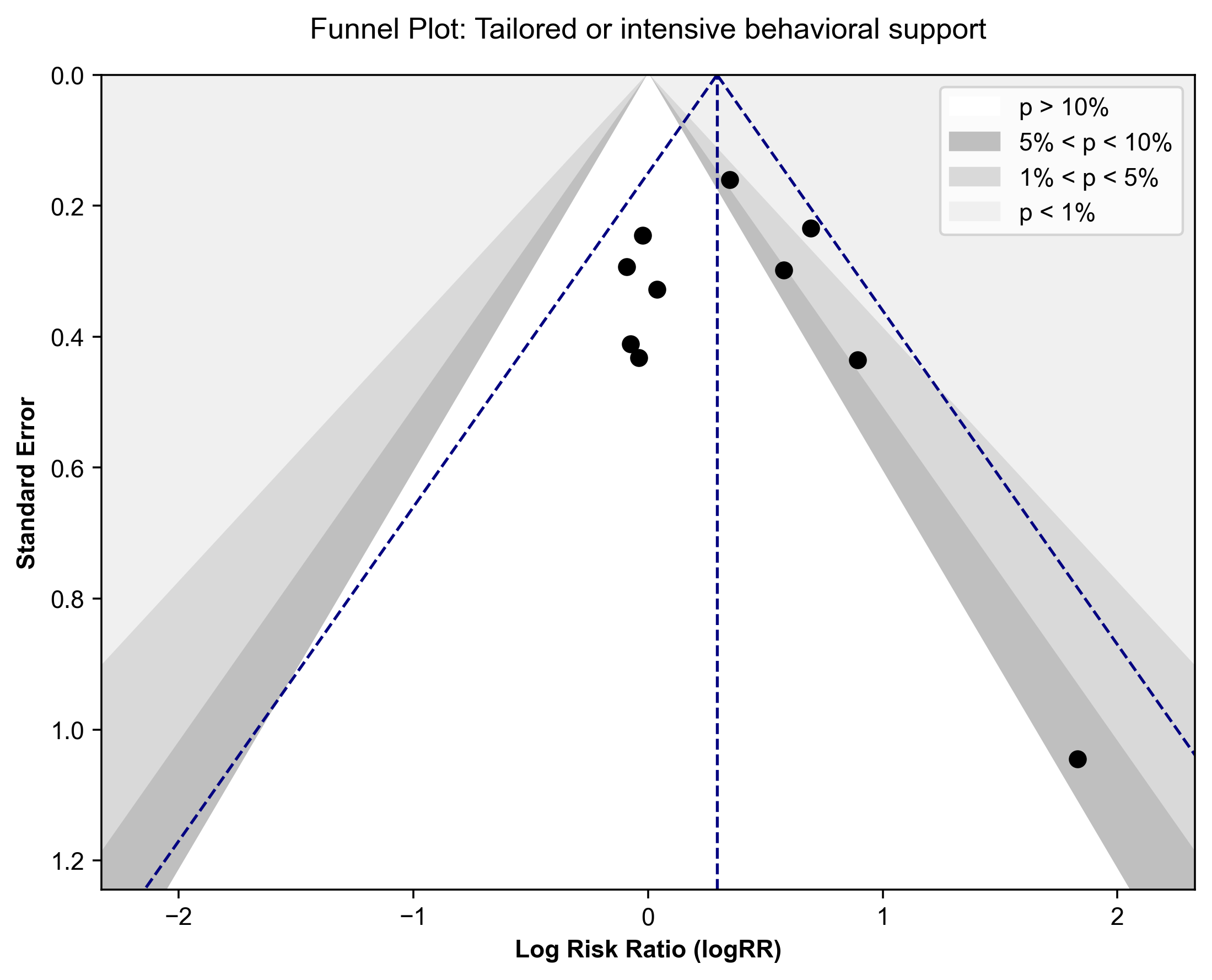

Supplement: S3 Fig — (TIF) [file pone.0350040.s012.tif]

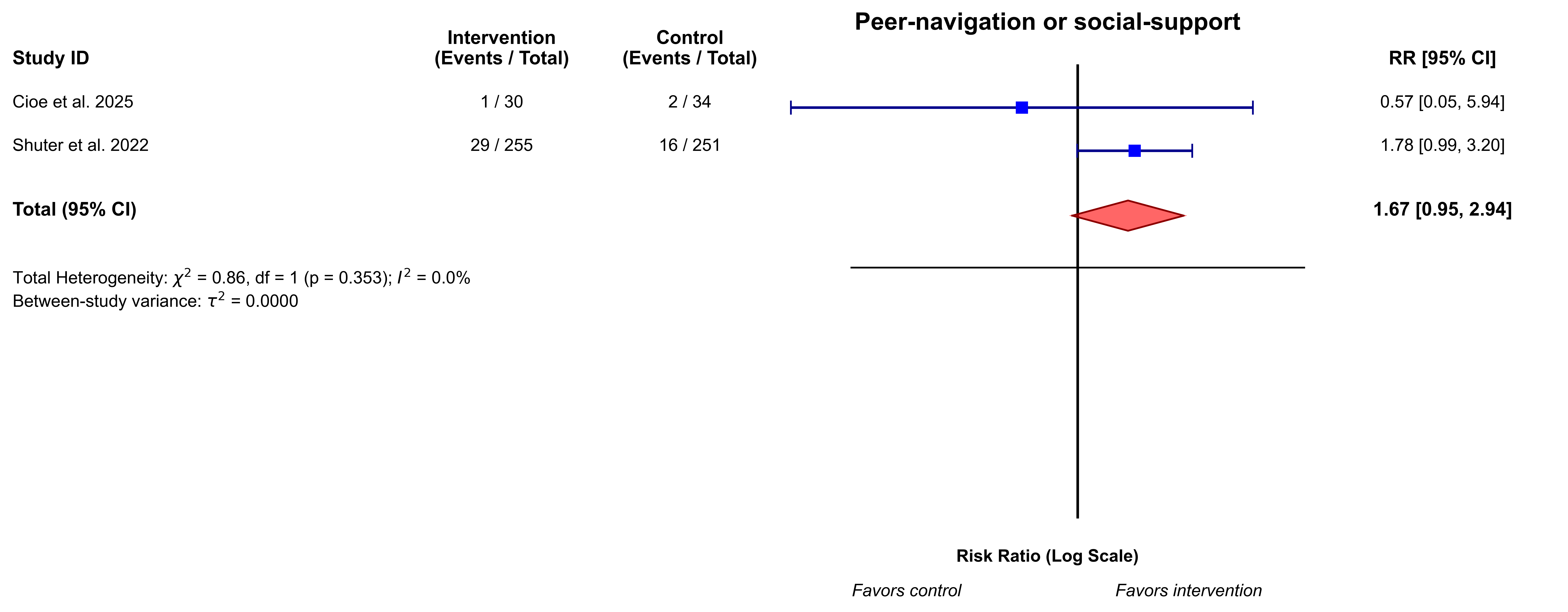

Supplement: S4 Fig — (TIF) [file pone.0350040.s013.tif]

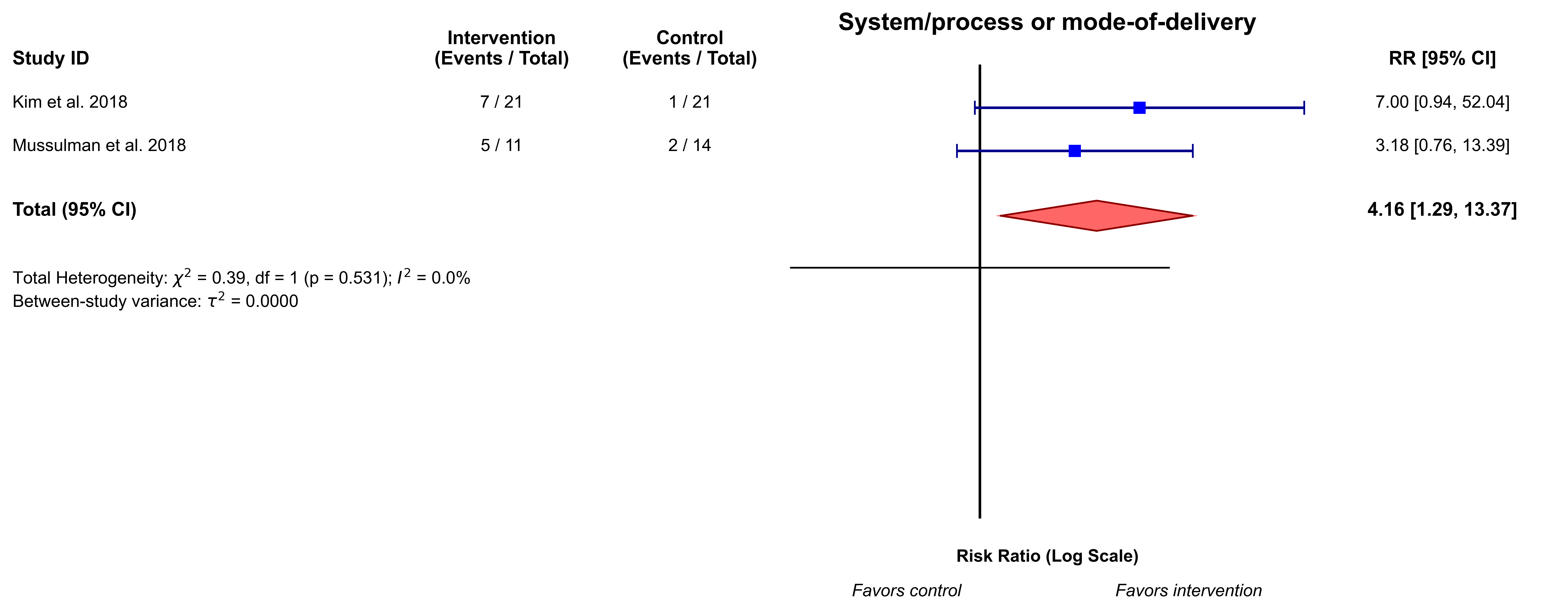

Supplement: S5 Fig — (TIF) [file pone.0350040.s014.tif]
