# Supplementary material for: Integrating smoking cessation into HIV care settings: A systematic review and meta-analysis of effectiveness and the evidence gap in cost-effectiveness
Source: PLoS One. 2026 Jul 30;21(7):e0350040. doi: 10.1371/journal.pone.0350040 (PMC13423040; doi:10.1371/journal.pone.0350040)
Supplement: Attachment — Submitted filename: Rebuttal letter.docx [file pone.0350040.s015.docx]

April 16, 2026

Rebuttal letter regarding submission PONE-D-26-16741

Dear Editor-in-Chief and Editors at PloS One Journal,

Please find enclosed the point-by-point responses for our re-submission of the manuscript entitled "Integrating smoking cessation into HIV care settings: A systematic review of the evidence on intervention effectiveness and cost-effectiveness" by Van Minh Nguyen, Thanh Hoang, Yesim Tozan, Mikael Svensson, Minh Van Hoang, and Nawi Ng.

In the previous decision (PONE-D-26-16741), we received the following feedback from the editorial team:

*“In particular, we have concerns about the analytical approaches for the meta-analysis. Incomplete or inappropriate statistical analyses can affect the generalizability and validity of the conclusions of a meta-analysis. It is important to identify and consider heterogeneity, the suitability of publication bias assessments, and the most appropriate statistical approach for the meta-analysis. In this case, we are concerned that one or more of these analyses is not conducted appropriately or without adequate justification.”*

We greatly appreciate this rigorous and constructive feedback. It highlighted several areas where our methodology and reporting needed to be brought into stricter alignment with the *Cochrane Handbook for Systematic Reviews of Interventions* and the GRADE framework. We have conducted a comprehensive methodological overhaul of our meta-analysis to address these concerns.

Below is a detailed, point-by-point summary of the revisions made to the manuscript and supplementary materials:

**1. The Most Appropriate Statistical Approach and Sensitivity Analyses**

- **Correction of Statistical Model:** We have replaced the contradictory "Mantel-Haenszel random-effects model" with the Cochrane-recommended **Generic Inverse-Variance method** for our random-effects model. To estimate between-study variance (Tau-squared), we utilized the iterative **Paule-Mandel (PM) estimator**, which is highly recommended for binary outcomes and small numbers of studies to reduce false precision.
- **Sensitivity Analyses for Small Subgroups:** To address the reviewer's concerns about the validity of random-effects models with very few studies, we instituted rigorous sensitivity analyses. We compared the primary PM Random-Effects model against a Fixed-Effect model and a conservative **Modified Hartung-Knapp-Sidik-Jonkman (HKSJ) model**. Where the HKSJ penalty caused the confidence intervals to widen and cross the line of no effect (e.g., in specific pharmacotherapy subgroups), we transparently reported this fragility in the Results and appropriately downgraded the GRADE certainty of evidence for Imprecision.

**2. Correction of Unit-of-Analysis Errors**

- In our previous draft, multiple intervention arms from a single study were calculated independently against a single control group, which introduced a unit-of-analysis error (double-counting the control group). We have rectified this. As detailed in the revised Methods, we now **pool data from multiple active intervention arms** to create a single, combined intervention group. This ensures all pairwise comparisons and data points in our forest and funnel plots are strictly independent.

**3. Identification and Consideration of Heterogeneity**

- **Comprehensive Reporting:** We have updated the Methods and Results to ensure that heterogeneity is assessed not just by I^2^, but by interpreting it alongside the magnitude and direction of treatment effects, the Tau-squared estimate, and the P-value from the Chi-squared test.

**4. Suitability of Publication Bias Assessments**

- **Formal Methodology Added:** We have thoroughly updated our Methods to specify how publication bias was formally assessed. We utilized **Contour-Enhanced Funnel Plots** and **Egger’s Regression Test** to evaluate asymmetry.
- **Application of the 10-Study Rule:** In strict accordance with Cochrane guidelines, we only applied these formal statistical tests and funnel plots to meta-analyses containing 10 or more independent studies (e.g., our behavioral support comparison) to ensure sufficient statistical power to distinguish true asymmetry from chance.

**5. Streamlined Reporting and Updated PRISMA**

- Finally, we have updated our PRISMA 2020 checklist to reflect our new methods (sensitivity analyses, subgroup analyses, etc.). We also streamlined the reporting of our GRADE assessments, providing concise summaries in the main text and *Summary of Findings* table (Table 4), while moving the highly granular, domain-by-domain Evidence Profiles to the Supplementary Materials.

We believe that these extensive revisions provide a methodologically robust meta-analysis that directly addresses all of the reviewers' concerns and significantly strengthens the validity of our conclusions.

Sincerely,

Van Minh Nguyen

University of Gothenburg, Sahlgrenska Academy

Institute of Medicine, School of Public Health and Community Medicine

Guldhedsgatan 5 A, plan 3, 413 20, Göteborg, Sweden

Email: van.minh.nguyen@gu.se/nmv@huph.edu.vn
