# Supplementary material for: Integrating smoking cessation into HIV care settings: A systematic review and meta-analysis of effectiveness and the evidence gap in cost-effectiveness
Source: PLoS One. 2026 Jul 30;21(7):e0350040. doi: 10.1371/journal.pone.0350040 (PMC13423040; doi:10.1371/journal.pone.0350040)
Supplement: Attachment — Submitted filename: Response_to_Reviewers.docx [file pone.0350040.s016.docx]

**Response to Reviewers**

**Manuscript PONE-D-26-19073 — PLOS ONE**

*Integrating smoking cessation into HIV care settings: a systematic review and meta-analysis of effectiveness and the evidence gap in cost-effectiveness*

Dear Dr. De Socio,

We thank you and the two reviewers for the careful and constructive assessment of our manuscript. The comments have substantially improved the paper. In response, we have reframed the review as a systematic review and meta-analysis, sharpened its rationale and contribution relative to the 2024 Cochrane review, condensed the Methods and Results and moved technical details to the Supporting Information, and moderated our interpretation throughout. We respond to each point below; reviewer comments are shown in italics, with our responses beneath. Line numbers refer to the revised manuscript. All changes are reflected in the revised manuscript and the marked-up (tracked-changes) version.

# Editor and journal requirements

**1.** *Ensure the manuscript meets PLOS ONE style requirements, including file naming.*

**Response.** The manuscript and Supporting Information have been reformatted to conform to the PLOS ONE style templates, and the submission files are named according to the journal's conventions.

**2.** *Provide a complete Data Availability Statement.*

**Response.** We confirm the following statement: “All data used in this study were extracted from previously published studies and are fully reported in the manuscript and its Supporting Information files.” This statement has also been entered in the submission form.

**3.** *Include captions for Supporting Information files at the end of the manuscript and update in-text citations accordingly.*

**Response.** A Supporting Information section listing all items with captions has been added at the end of the manuscript (S1 Checklist, S1–S3 Text, S1–S5 Tables, and S1–S5 Figures), and every item is now cited in the text using the PLOS naming convention (Lines 458–475).

**4.** *Evaluate any reviewer-recommended citations for relevance.*

**Response.** We have added the methodological citations requested by Reviewer 2 (see Reviewer 2, specific comments 1 and 2).

# Reviewer 1

We thank the reviewer for recognizing the public-health importance of the topic, the focused objective, the structured PRISMA-based methodology, and the breadth of the search, and for the constructive editorial guidance. We have addressed each point below.

## General comments

**1.1** *The stated aim addresses both effectiveness and cost-effectiveness, but the Results report that no cost or cost-effectiveness studies were identified, creating a mismatch between the title/aim and the evidence retrieved.*

**Response.** We agree and have reframed the review so that cost-effectiveness is presented as an identified evidence gap rather than a delivered result. The title is now “Integrating smoking cessation into HIV care settings: a systematic review and meta-analysis of effectiveness and the evidence gap in cost-effectiveness.” The aim, abstract, and Methods now state that economic evaluations were sought but none were found, and this absence is reported explicitly as a key gap in the evidence base (In the title Lines 1–3, and in Lines 85–86).

**1.2** *The review combines randomized, non-randomized, cohort, and single-arm studies; the manuscript should explain why such heterogeneous designs were combined, what question each answers, and how this affects causal interpretation.*

**Response.** We have clarified the rationale in the Methods (Lines 147–150). Different study designs were included to address complementary research questions. Randomized controlled trials provide the strongest evidence on the causal effects of interventions under controlled conditions and therefore form the basis of the meta-analyses, whereas non-randomized, cohort, and single-arm studies were included to capture real-world implementation, longer-term outcomes, feasibility, and contextual variation. Given these methodological differences, non-randomized studies were not pooled quantitatively with the trials but were synthesized narratively to contextualize the trial findings. We explicitly acknowledge that causal inference rests primarily on the randomized evidence, whereas findings from non-randomized designs are interpreted with greater caution owing to potential confounding and bias, with certainty graded accordingly under the GRADE framework.

**1.3** *Several methods passages are dense — pooling of multiple intervention arms, factorial trials, SMART designs, and the choice of random-effects estimator — and need clearer wording for an international audience.*

**Response.** We have rewritten these passages in plainer language and relocated the technical mechanics to S1 Text. The main-text Methods now retain a concise summary that states the pooling rules directly (multi-arm trials pooled against the shared control; factorial main effects isolated across the other factor; head-to-head and SMART trials informing direct comparative analyses), with the full detail in S1 Text (Lines 150–155).

**1.4** *The manuscript should avoid sounding stronger than the evidence allows, given that only a few LMIC trials were identified and no economic evaluations were found.*

**Response.** We have moderated the interpretation substantively, not only at the level of wording. Claims are now tied explicitly to the GRADE certainty levels; the LMIC trials are described as establishing feasibility and providing preliminary efficacy data, with their small number and mixed results explicitly noted; the behavioral-support finding is framed as the effect of tailored, intensive intervention packages as a class rather than of any isolated component; and the limitations, including the predominance of high-income data and the absence of economic evaluations, are integrated into the interpretation. The Discussion, Conclusions, and Abstract have been revised accordingly (Lines 408–411, 440–447).

**1.5** *The Results contain many details that read as extracted notes rather than a synthesized narrative, making the central comparative findings difficult to identify.*

**Response.** We have substantially condensed and restructured the Results into a synthesized narrative organized around the main comparisons. Exhaustive per-study descriptions of interventions, controls, and designs have been relocated to S2 Table, S2 Text, S3 Text and Table 3, and each subsection now leads with the comparative finding (Lines 170–350).

# Reviewer 2

We thank the reviewer for the thoughtful critique, particularly regarding the rationale for the review's scope and its relationship to the 2024 Cochrane review. These comments prompted a substantial reframing that we believe has clarified the paper's contribution.

## General comments

**2.1** *It is unclear why a review this broad is necessary two years after the Cochrane review, and why high-income countries are included; no case is made for the evidence gaps beyond the lack of LMIC studies.*

**Response.** We have revised the Introduction to state the specific evidence gaps and the rationale for the global scope (Lines 80–87). The review articulates four advances over the 2024 Cochrane review: (i) the first pooled evidence for bupropion in people living with HIV; (ii) the inclusion of non-randomized designs alongside randomized trials; (iii) formal GRADE certainty ratings across intervention types; and (iv) the first synthesis to incorporate multiple LMIC trials. The high-income literature is retained because it provides the comparator and the statistical power for the pooled estimates, while the LMIC evidence is now reported distinctly.

**2.2** *Have the conclusions changed since the Cochrane review? If not, why conduct another global review?*

**Response.** They have changed in important respects, and we now make this explicit in the Discussion (Lines 352–361). The present review provides the first efficacy evidence for bupropion (which the Cochrane review found to be untested); it uses the published data from the South African trial, which the Cochrane review could access only as unpublished data; it draws on larger pooled samples; and it identifies a benefit for tailored, intensive behavioral support. We frame the behavioral finding as complementary to, rather than contradictory of, the Cochrane review (see comment 2.3).

**2.3** *The evidence synthesis appears to combine HIC and LMIC data, so it is not clear how it helps the LMIC evidence gap; consider limiting the review to LMICs, or synthesizing LMIC evidence separately and comparing it with the HIC-dominated Cochrane review.*

**Response.** We have adopted the reviewer's second suggestion. The LMIC evidence is now reported distinctly and interpreted against the high-income literature (Lines 381–390). We retained the global scope rather than restricting to LMICs because only three LMIC trials were identified, which is too few to support a standalone synthesis, and because the comparison with the larger high-income evidence base is itself informative. On the behavioral-support finding specifically, the present review and the Cochrane review addressed different questions: the Cochrane review compared behavioral support against brief advice with pharmacotherapy held constant, estimating the incremental effect of the behavioral component, whereas we asked whether tailored, intensive intervention packages outperform brief or standard care. Both questions are legitimate, and the two sets of findings are complementary rather than contradictory.

**2.4** *Given the limited evidence, consider relaxing some inclusion/exclusion criteria — for example, the requirement for a minimum 6-month follow-up.*

**Response.** We considered this carefully and elected to retain the ≥6-month criterion on methodological grounds. It aligns with the Russell Standard for long-term cessation and preserves direct comparability with the Cochrane review, which applied the same threshold (Lines 102–104). A shorter follow-up would capture early, often transient abstinence rather than the sustained cessation that is the clinically meaningful outcome for people living with HIV. To acknowledge the near-miss LMIC evidence, we now summarize the LMIC studies excluded for shorter follow-up in S3 Table, so that this emerging activity is visible without compromising the primary outcome definition.

**2.5** *A systematic review does not pool results, whereas a meta-analysis does. Since results were pooled, should this not be a meta-analysis?*

**Response.** We agree. As the review pools effect estimates with random-effects models and reports GRADE certainty, we have relabeled it a “systematic review and meta-analysis” throughout, including in the title, and have confirmed reporting against the PRISMA 2020 statement (Lines 1–3).

**2.6** *The paper is very long; the Methods and Results are overwhelming, and some material should be moved to the supplement so the manuscript is more manageable.*

**Response.** We have substantially condensed both sections. Technical methodological detail has been moved to S1 Text, and exhaustive per-study descriptions to S2 Table and Table 3. The Results now present a synthesized narrative organized around the main comparisons, and the GRADE summary of findings (Table 4) carries the headline results (Lines 145–350). The total word count has reduced significantly, from more than 12,000 words to around 9,000 words.

## Specific comments

**1.** *Provide a methodological citation for the Paule–Mandel estimator (line 153).*

**Response.** Added. We now cite the original description of the Paule–Mandel estimator (Line 158).

**2.** *Provide a methodological citation for the HKSJ model (line 156).*

**Response.** Added. We now cite the Hartung–Knapp–Sidik–Jonkman method (Line 160).

**3.** *“were” should be “was” (line 170).*

**Response.** Corrected (Line 164).

**4.** *The GRADE citations can do the heavy lifting; the domains can simply be stated (lines 170–189).*

**Response.** We have trimmed this passage to state the GRADE domains with the supporting citations, in line with the reviewer's suggestion. The full domain definitions, including the standard GRADE conventions for assessing publication bias (restricted to comparisons with ≥10 studies) and for upgrading certainty on the basis of a large effect, are provided in S1 Text (Lines 164–169).

**5.** *It is unclear what “These” refers to, since the previous sentence discussed only four articles (line 208).*

**Response.** We have clarified the passage. The text now states that the LMIC evidence comprises three randomized controlled trials reported across four articles, with Elf et al. and its re-treatment extension Keke et al. constituting the same South African trial. The ambiguous referent has been removed (Lines 191–194).

We hope these revisions address the reviewers' comments fully and are happy to make any further changes the editor considers necessary. We thank the reviewers again for their careful reading, which has materially improved the manuscript.

Yours sincerely,

Van Minh Nguyen, on behalf of all authors
