# Supplementary material for: Integrating smoking cessation into HIV care settings: A systematic review and meta-analysis of effectiveness and the evidence gap in cost-effectiveness
Source: PLoS One. 2026 Jul 30;21(7):e0350040. doi: 10.1371/journal.pone.0350040 (PMC13423040; doi:10.1371/journal.pone.0350040)
Supplement: Peer review history

# Author comment: Author response to Decision Letter 0

**DOI:** 10.1371/journal.pone.0350040.r001

21 Apr 2026

Attachment Submitted filename: Rebuttal letter.docx

# Peer review: Decision Letter 0

**DOI:** 10.1371/journal.pone.0350040.r002

**Authors:** Giuseppe De Socio

15 Jun 2026

PONE-D-26-19073

Integrating smoking cessation into HIV care settings: A systematic review of the evidence on intervention effectiveness and cost-effectiveness

PLOS One

Dear Dr. Nguyen,

Thank you for submitting your manuscript to PLOS ONE. After careful consideration, we feel that it has merit but does not fully meet PLOS ONE’s publication criteria as it currently stands. Therefore, we invite you to submit a revised version of the manuscript that addresses the points raised during the review process.

Please submit your revised manuscript by Jul 30 2026 11:59PM. If you will need more time than this to complete your revisions, please reply to this message or contact the journal office at plosone@plos.org. When you're ready to submit your revision, log on to https://www.editorialmanager.com/pone/ and select the 'Submissions Needing Revision' folder to locate your manuscript file.

Please include the following items when submitting your revised manuscript:

- A letter that responds to each point raised by the academic editor and reviewer(s). You should upload this letter as a separate file labeled 'Response to Reviewers'.
- A marked-up copy of your manuscript that highlights changes made to the original version. You should upload this as a separate file labeled 'Revised Manuscript with Track Changes'.
- An unmarked version of your revised paper without tracked changes. You should upload this as a separate file labeled 'Manuscript'.

If you would like to make changes to your financial disclosure, please include your updated statement in your cover letter. Guidelines for resubmitting your figure files are available below the reviewer comments at the end of this letter.

If applicable, we recommend that you deposit your laboratory protocols in protocols.io to enhance the reproducibility of your results. Protocols.io assigns your protocol its own identifier (DOI) so that it can be cited independently in the future. For instructions see: https://journals.plos.org/plosone/s/submission-guidelines#loc-laboratory-protocols. Additionally, PLOS ONE offers an option for publishing peer-reviewed Lab Protocol articles, which describe protocols hosted on protocols.io. Read more information on sharing protocols at https://plos.org/protocols?utm_medium=editorial-email&utm_source=authorletters&utm_campaign=protocols.

As the corresponding author, your ORCID iD is verified in the submission system and will appear in the published article. PLOS supports the use of ORCID, and we encourage all coauthors to register for an ORCID iD and use it as well. Please encourage your coauthors to verify their ORCID iD within the submission system before final acceptance, as unverified ORCID iDs will not appear in the published article. *Only*  the individual author can complete the verification step; PLOS staff *cannot*  verify ORCID iDs on behalf of authors.

We look forward to receiving your revised manuscript.

Kind regards,

Giuseppe Vittorio De Socio, MD, PhD

Academic Editor

PLOS One

Journal Requirements:

When submitting your revision, we need you to address these additional requirements.

1.Please ensure that your manuscript meets PLOS ONE's style requirements, including those for file naming. The PLOS ONE style templates can be found at

https://journals.plos.org/plosone/s/file?id=wjVg/PLOSOne_formatting_sample_main_body.pdf and

https://journals.plos.org/plosone/s/file?id=ba62/PLOSOne_formatting_sample_title_authors_affiliations.pdf

2. Please provide a complete Data Availability Statement in the submission form, ensuring you include all necessary access information or a reason for why you are unable to make your data freely accessible. If your research concerns only data provided within your submission, please write "All data are in the manuscript and/or supporting information files" as your Data Availability Statement.

3. Please include captions for your Supporting Information files at the end of your manuscript, and update any in-text citations to match accordingly. Please see our Supporting Information guidelines for more information: http://journals.plos.org/plosone/s/supporting-information.

4. If the reviewer comments include a recommendation to cite specific previously published works, please review and evaluate these publications to determine whether they are relevant and should be cited. There is no requirement to cite these works unless the editor has indicated otherwise.

[Note: HTML markup is below. Please do not edit.]

Reviewers' comments:

Reviewer's Responses to Questions

**Comments to the Author**

1. Is the manuscript technically sound, and do the data support the conclusions?

The manuscript must describe a technically sound piece of scientific research with data that supports the conclusions. Experiments must have been conducted rigorously, with appropriate controls, replication, and sample sizes. The conclusions must be drawn appropriately based on the data presented.

Reviewer #1: Partly

Reviewer #2: No

**********

2. Has the statistical analysis been performed appropriately and rigorously?

Reviewer #1: I Don't Know

Reviewer #2: Yes

**********

3. Have the authors made all data underlying the findings in their manuscript fully available?

The PLOS Data policy requires authors to make all data underlying the findings described in their manuscript fully available without restriction, with rare exception (please refer to the Data Availability Statement in the manuscript PDF file). The data should be provided as part of the manuscript or its supporting information, or deposited to a public repository. For example, in addition to summary statistics, the data points behind means, medians and variance measures should be available. If there are restrictions on publicly sharing data—e.g. participant privacy or use of data from a third party—those must be specified.

Reviewer #1: Yes

Reviewer #2: Yes

**********

4. Is the manuscript presented in an intelligible fashion and written in standard English?

PLOS ONE does not copyedit accepted manuscripts, so the language in submitted articles must be clear, correct, and unambiguous. Any typographical or grammatical errors should be corrected at revision, so please note any specific errors here.

Reviewer #1: Yes

Reviewer #2: Yes

**********

5. Review Comments to the Author

Please use the space provided to explain your answers to the questions above. You may also include additional comments for the author, including concerns about dual publication, research ethics, or publication ethics. (Please upload your review as an attachment if it exceeds 20,000 characters)

Reviewer #1: The paper has several clear strengths. It frames the public health importance of smoking in people living with HIV convincingly, defines a focused review objective, uses a structured PRISMA-based methodology, and documents a broad database search performed in March 2026. The manuscript also appears ambitious in scope because it includes effectiveness, costs, and cost-effectiveness, and it adds newer studies from low- and middle-income settings that were underrepresented in prior reviews.

At the same time, the draft would benefit from substantial editorial revision before submission. The main issues concern, consistency of terminology, clarity of methods, tighter interpretation of findings, and stronger alignment between the stated objectives and the results actually obtained.

The stated aim is to provide up-to-date evidence on both effectiveness and cost-effectiveness of smoking cessation interventions for people living with HIV. However, the Results section explicitly states that no studies evaluating cost or cost-effectiveness were identified, which creates a mismatch between the title/aim and the actual evidence base retrieved

The review combines randomized trials, non-randomized studies, cohort studies, and apparently single-arm intervention studies. This broad inclusion can be justified, but the manuscript should explain more explicitly why such heterogeneous designs were combined, what question each design answers, and how this affects causal interpretation.

Several methods passages are difficult to follow because the syntax is dense. For example, the sections on pooling multiple intervention arms, factorial trials, SMART designs, and choice of random-effects estimator are methodologically relevant but need much clearer wording for an international journal audience.

The draft repeatedly emphasizes that it expands the literature and addresses major gaps, especially in low- and middle-income countries. That may be true, but the paper should avoid sounding stronger than the evidence allows because only four LMIC randomized trials were identified and no economic evaluations were found.

The Results section contains many details that read like extracted notes rather than a synthesized narrative. For instance, descriptions of individual interventions, control conditions, and study designs are often exhaustive, but the reader still has difficulty identifying the central comparative findings.

Reviewer #2: General comments:

This manuscript presents results for a systematic review (SR) on smoking cessation interventions in people living with HIV (PLWH).

My main criticism of this manuscript is that I don't entirely follow why a SR this broad is necessary just two years after a Cochrane review was published. I think the justification provided in lines 70-77 for why some sort of evidence synthesis is needed for low- and middle-income countries. That is very compelling and I found it interesting that multiple studies have been published since the Cochrane review was completed. But, I don't understand why the high-income countries (HICs) are included in this SR. The thesis statement for this manuscript appears to be in line 80 ("...address these evidence gaps..."), but no case has been made for what those evidence gaps are beyond the lack of LMIC studies.

Said differently, why are HICs included? Have the conclusions changed since the Cochrane review? If so, why do another "global" SR?

In addition, the results evidence synthesis appears to happen together for HICs and LMICs, so it's not entirely clear to me how this is helping the evidence gap for LMICs. Maybe your argument is that a greater proportion of the evidence is coming from LMICs. That's a fair point, but it would seem to me to that synthesizing the evidence from LMICs and then comparing that to the HIC-dominated Cochrane review would be more compelling since the results from LMIC would be reported on its own.

My recommendation is to limit this SR to only LMICs since that is the identified evidence gap. I apologize if I have missed something major with your manuscript, but that seems to me to be the major contribution to the literature. Feel free to correct me if I am wrong. I also think since you are doing an SR and that there is not that much information that exists, you might consider being more lenient with some of your inclusion/exclusion criteria. For instance, on lines 92-93, you mentioned that studies required a minimum 6-month follow-up. You may want to relax that somewhat if there are more studies that could be included.

Also, my understanding is that a SR doesn't pool results together and a meta-analysis does. Since you pooled results, shouldn't this be a meta-analysis?

Finally, this is a very long paper. I got lost in the methods and found myself questioning whether all of the methods are actually relevant. You are going to lose readers or they are just going to skip sizeable sections of this. I urge you to think very carefully about the methods and results sections because they are overwhelming. I strongly encourage you to shift some of this information to the supplement so that the manuscript is more manageable to the majority of readers. Those who want all the gory details can go to the supplement.

Specific comments:

1. (line 153) Please provide a methodological citation for the Paule-Mandel estimator.

2. (line 156) Please provide a methodological citation for the HKSJ model.

3. (line 170) "were" should be "was".

4. (lines 170-189) I think you can rely on the GRADE citations to do the heavy-lifting here and you can just state the domains.

5. (line 208) I don't know what "These" is referring to since the previous sentence only discussed four articles.

**********

6. PLOS authors have the option to publish the peer review history of their article (what does this mean?). If published, this will include your full peer review and any attached files.

If you choose “no”, your identity will remain anonymous but your review may still be made public.

**Do you want your identity to be public for this peer review?**  For information about this choice, including consent withdrawal, please see our Privacy Policy.

Reviewer #1: No

Reviewer #2: No

**********

[NOTE: If reviewer comments were submitted as an attachment file, they will be attached to this email and accessible via the submission site. Please log into your account, locate the manuscript record, and check for the action link "View Attachments". If this link does not appear, there are no attachment files.]

To ensure your figures meet our technical requirements, please review our figure guidelines: https://journals.plos.org/plosone/s/figures

You may also use PLOS’s free figure tool, NAAS, to help you prepare publication quality figures: https://journals.plos.org/plosone/s/figures#loc-tools-for-figure-preparation.

NAAS will assess whether your figures meet our technical requirements by comparing each figure against our figure specifications.

# Author comment: Author response to Decision Letter 1

**DOI:** 10.1371/journal.pone.0350040.r003

10 Jul 2026

Please see the attached Response to Reviewers for a point-by-point response to each comment, with line references to the revised manuscript.

In brief, we have reframed the study as a systematic review and meta-analysis and now present cost-effectiveness as an identified evidence gap, as reflected in the revised title. We have clarified the review's rationale and contribution relative to the 2024 Cochrane review and now report the LMIC evidence distinctly. The Methods and Results have been substantially condensed, with technical detail moved to the Supporting Information, and the interpretation has been moderated throughout.

Attachment Submitted filename: Response_to_Reviewers.docx

# Editor report: Decision Letter 1

**DOI:** 10.1371/journal.pone.0350040.r004

**Authors:** Giuseppe De Socio

14 Jul 2026

Integrating smoking cessation into HIV care settings: a systematic review and meta-analysis of effectiveness and the evidence gap in cost-effectiveness

PONE-D-26-19073R1

Dear Dr. Nguyen,

We’re pleased to inform you that your manuscript has been judged scientifically suitable for publication and will be formally accepted for publication once it meets all outstanding technical requirements.

Within one week, you’ll receive an e-mail detailing the required amendments. When these have been addressed, you’ll receive a formal acceptance letter and your manuscript will be scheduled for publication.

An invoice will be generated when your article is formally accepted. Please note, if your institution has a publishing partnership with PLOS and your article meets the relevant criteria, all or part of your publication costs will be covered. Please make sure your user information is up-to-date by logging into Editorial Manager at Editorial Manager® and clicking the ‘Update My Information' link at the top of the page. For questions related to billing, please contact billing support.

If your institution or institutions have a press office, please notify them about your upcoming paper to help maximize its impact. If they’ll be preparing press materials, please inform our press team as soon as possible -- no later than 48 hours after receiving the formal acceptance. Your manuscript will remain under strict press embargo until 2 pm Eastern Time on the date of publication. For more information, please contact onepress@plos.org.

Kind regards,

Giuseppe Vittorio De Socio, MD, PhD

Academic Editor

PLOS One

Additional Editor Comments (optional):

Reviewers' comments:

# Editor report: Acceptance letter

**DOI:** 10.1371/journal.pone.0350040.r005

**Authors:** Giuseppe De Socio

PONE-D-26-19073R1

PLOS One

Dear Dr. Nguyen,

I'm pleased to inform you that your manuscript has been deemed suitable for publication in PLOS One. Congratulations! Your manuscript is now being handed over to our production team.

At this stage, our production department will prepare your paper for publication. This includes ensuring the following:

* All references, tables, and figures are properly cited

* All relevant supporting information is included in the manuscript submission,

* There are no issues that prevent the paper from being properly typeset

You will receive further instructions from the production team, including instructions on how to review your proof when it is ready. Please keep in mind that we are working through a large volume of accepted articles, so please give us a few days to review your paper and let you know the next and final steps.

Lastly, if your institution or institutions have a press office, please let them know about your upcoming paper now to help maximize its impact. If they'll be preparing press materials, please inform our press team within the next 48 hours. Your manuscript will remain under strict press embargo until 2 pm Eastern Time on the date of publication. For more information, please contact onepress@plos.org.

You will receive an invoice from PLOS for your publication fee after your manuscript has reached the completed accept phase. If you receive an email requesting payment before acceptance or for any other service, this may be a phishing scheme. Learn how to identify phishing emails and protect your accounts at https://explore.plos.org/phishing.

If we can help with anything else, please email us at customercare@plos.org.

Thank you for submitting your work to PLOS ONE and supporting open access.

Kind regards,

PLOS ONE Editorial Office Staff

on behalf of

prof. Giuseppe Vittorio De Socio

Academic Editor

PLOS One
